# Phylodynamics of the Emergence of Influenza Viruses after Cross-Species Transmission

**DOI:** 10.1371/journal.pone.0082486

**Published:** 2013-12-16

**Authors:** Leila Rahnama, Stéphane Aris-Brosou

**Affiliations:** 1 Department of Biology, Center for Advanced Research in Environmental Genomics, University of Ottawa, Ottawa, Ontario, Canada; 2 Department of Mathematics and Statistics, University of Ottawa, Ottawa, Ontario, Canada; University of Muenster, Germany

## Abstract

Human populations are constantly exposed to emerging pathogens such as influenza A viruses that result from cross-species transmissions. Generally these sporadic events are evolutionary dead-ends, but occasionally, viruses establish themselves in a new host that offers a novel genomic context to which the virus must adjust to avoid attenuation. However, the dynamics of this process are unknown. Here we present a novel method to characterize the time it takes to G+C composition at third codon positions (GC3 content) of influenza viruses to adjust to that of a new host. We compare the inferred dynamics in two subtypes, H1N1 and H3N2, based on complete genomes of viruses circulating in humans, swine and birds between 1900–2009. Our results suggest that both subtypes have the same fast-adjusting genes, which are not necessarily those with the highest absolute rates of evolution, but those with the most relaxed selective pressures. Our analyses reveal that NA and NS2 genes adjust the fastest to a new host and that selective pressures of H3N2 viruses are relaxed faster than for H1N1. The asymmetric nature of these processes suggests that viruses with the greatest adjustment potential to humans are coming from both birds and swine for H3N2, but only from birds for H1N1.

## Introduction

Influenza outbreaks remain a major life-threatening condition that generates a serious burden on public health, accompanied by acute economic losses on a global scale [1]. The etiological agent of these outbreaks, the influenza A virus, circulates in a relatively wide range of hosts such as humans, pigs and birds [2], with wild waterfowl usually considered to be the reservoir host [1]. Because of the physical proximity of these different hosts, spillovers occur quite frequently. Although past pandemics in the human population were caused by such transmissions from one animal species to another [3], [4], host changes rarely lead to epidemics in the new host [5]. The majority of these cross-species transmissions actually ends up as evolutionary dead-ends for the virus [6], but they occasionally lead to stable lineages that establish themselves in the new host [7].

A key requirement for the emergence of such stable viral lineages is circumventing host restriction [8]. Influenza A viruses are known to have mechanisms in place that limit cross-species transmission, since for instance all viral subtypes are not found in all potential hosts. Recent studies have shed some light on the nature of these mechanisms. Specifically, out of up to 14 protein-coding genes distributed on eight single-stranded negative-sense RNA segments, the three polymerase subunits (PB2, PB1 and PA) and the nucleoprotein (NP) that form the ribonucleoprotein (RNP) have been argued to be closely involved in host restriction [2]. Similarly, the two surface antigenes hemagglutinin (HA) and neuraminidase (NA) influence viral host restriction [9]. Out of the six other products, three of them, PB1-F2 [10], PB1-N40 [11] and PA-X [12], are not found in all viruses and the four others are splice variants matrix proteins (M2/M1 and M42 in some strains [13]) and nonstructural proteins (NS2/NS1). Because both M1 and NS2 are involved in RNP transport out of the nucleus, it could also be argued that they need to be adapted to recognize the host machinery, and therefore that they participate in host restriction. To some extent then, previous studies have shown that almost all influenza A genes, when studied independently, are involved in host restriction. In a pioneering study, dos Reis *et al.* focused on the sites undergoing changes in selective pressures during cross-species transmission to identify host-specific patterns of adaptation across the genome of H1N1 viruses [14]. While their approach led them to discard 93% of the sites prior to analysis (as no evidence of host adaptation could be found at these sites), hereby reducing a whole-genome analysis to 294 amino acid sites, it can be posited that a change of host leaves a more pervasive signature across the entire viral genome. For instance, codon deoptimization, where sub-optimal codons replace the original codons, has been suggested as a vaccine-development strategy [15]. Conversely, if viral hosts have different codon biases, it can be expected that a host change will affect viral codon usage, and therefore the G+C composition at third codon positions (GC3) of all the viral genes. As previous studies have shown that influenza viruses do indeed exhibit host-specific GC3 contents ( *e.g.*, [16]), these viruses must be undergoing a GC3 *adjustment* after a host change – we here use the phrase of *viral adjustment* to describe this process, as GC3 change following a host change is not necessarily adaptive [17]. However, the dynamics of viral adjustment are completely unknown. In particular, it is unknown (i) if this process depends on the direction of host change, that is whether the virus leaps e.g. from an avian to a human host or from human to swine, (ii) if this process depends on the direction of host change, or (iii) if there is some variation among influenza A subtypes.

To address these questions, we developed a novel procedure to estimate the dynamics of the emergence of stable influenza A lineages following a cross-species transmission. Based on a phylogenetic approach, we reconstructed the history of both host and GC3 changes in the two most human-prevalent influenza A subtypes, H1N1 and H3N2, focusing on three hosts in which both of these subtypes have established themselves: human, avian and swine. With the analysis of almost 100 years of complete genomes collected in North America, we show that two genes, NA and NS2, adjust to a new host relatively quickly. We also show that the adjustment process is asymmetric among hosts, with viruses of avian origin adjusting the fastest. Finally, while the ranking of fast-adjusting genes is the same for both H1N1 and H3N2 subtypes, selective constraints of H3N2 are relaxed faster than for H1N1 viruses.

## Results and Discussion

### Sequence clustering

In order to estimate viral adjustment times in influenza A viruses after a host change, we retrieved the sequences of *complete* genomes of H1N1 and H3N2 subtypes from the Influenza Virus Resource [18]. We specifically downloaded *all* the genomes collected in North America (Mexico, the USA and Canada) between 1900 and 2009. Only one pandemic H1N1/2009 genome was included in this study [19]. This lead to an average of 1916 H1N1 and 1050 H3N2 sequences per gene.

After alignment, the size of the data sets was reduced to make them amendable to the Bayesian relaxed molecular clock analyses. Pairwise genetic distances were computed and clustered with the nearest neighbor algorithm; clusters of sequences similar at the 99% level were formed and a sequence representative of each cluster was drawn (see Methods for details and constraints). This clustering reduced the size of the data to more manageable numbers with an average of 75 H1N1 and 43 H3N2 sequences (Table S1 in File S1). These data sets therefore stand as representative samples of the exhaustive whole-genome diversity deposited in GenBank (as of January 2010). This reduction step affects the hypothesis underlying the coalescent process used as a prior distribution in the estimation of divergence times used below. However, since we (i) did not attempt to reconstruct ancestral demographics (viral incidence), (ii) used the same process to analyze both subtypes and (iii) expected that most adjustment periods did not occur following recent host changes, this reduction step is unlikely to bias the comparison of adjustment dynamics of H1N1 and H3N2 viruses.

### H1N1 and H3N2 subtypes evolve with extensive reassortment

Under this general framework, we reconstructed dated phylogenetic trees for all ten 'canonical' protein-coding genes of influenza viruses [20], [21] of the H1N1 (Fig. S1-S10 in File S1) and H3N2 subtypes (Fig. S11-S20 in File S1) under a relaxed molecular clock [22]. Note that we assumed a single (time-homogeneous) model of evolution instead of using nonhomogeneous models [23]; this choice could potentially impact the estimated trees, but a number of empirical studies have now shown that this concern may not be warranted ( *e.g.*, [4], [7]). Because the natural host of influenza viruses is considered to be avian [24], we expected that bird viruses would diverge first in all estimated trees. We also expected to find similar phylogenies for all ten genes within a given subtype, as the data come from the same individual viruses. However, the trees estimated here show a variety of scenarios, all with a posterior probability of 1 at the root node. Only PB2 and PA consistently show an avian-first split across the two subtypes, along with NP in H1N1 and NA and NS2 in H3N2 subtypes (Fig. S1-S20 in File S1). Known reassortment events are also recovered here, as in the case of A/Saskatchewan/5131/2009(H1N1), one of the two ''H1N1_Canada_Human_2009'' genomes in Fig. S1-S10 in File S1, which is a reassortant virus for which: (i) HA and NA are derived from the non-pandemic A/Brisbane/59/2007 human virus, as seen in Fig. S4 and S6 (File S1), (ii) PB2, PB1, PA, NP, M and NS are of swine origin (Fig. S1-S3, S5 and S7-S10 in File S1) and (iii) that this virus emerged during the late 1990's; all these results are consistent with the original study [19], which therefore suggests that our results are not data-dependent. These results nonetheless highlight that extensive amounts of reassortment (exchange of RNA segments between viruses) exist, at least within each subtype.

### Some genes evolve faster in H3N2 than in H1N1

**Figure 1:**
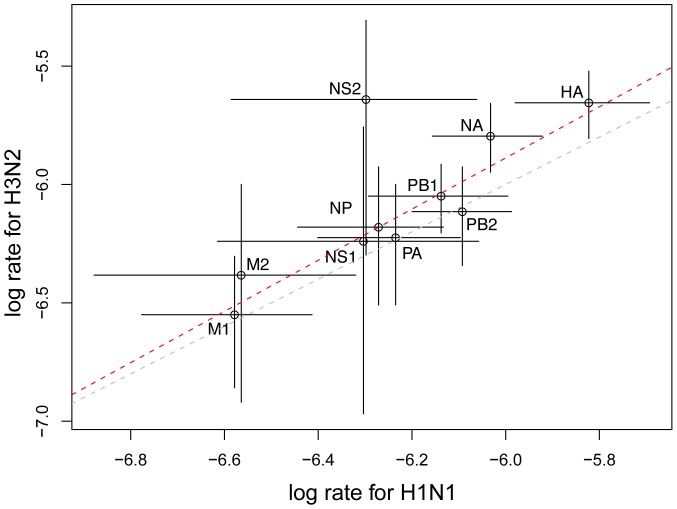
Posterior mean rates of evolution of H3N2 *vs.* H1N1 viruses. Results are shown on a 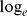-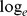 scale (in substitutions/site/year). The gray line represents the first bisector (line of equation 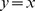), while the red line represents the linear fit to the data. Bars: limits of the 95% Highest Posterior Densities.

A by-product of the relaxed molecular clock models used here is the estimation of gene-specific absolute rates of evolution. Figure 1 shows that these rates are systematically larger for H3N2 than for H1N1 viruses, with a genome-wide average of 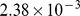 (SEM 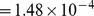) and 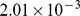 (SEM 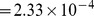) substitutions/site/year, respectively, but not significantly so (test on the intercept: 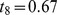, 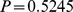). These estimates are very close to those previously reported [25] or with earlier knowledge of relative rates of evolution of H3N2 and H1N1 viruses [26]. The rate difference between the two subtypes appears to be significant (at the 5% level) only for three genes (HA, NA and NS2; Fig. 1). Because H3N2 has been the dominant subtype in human populations for the 40 years preceding 2009, it can be posited that these genes are under stronger selective pressure than in H1N1 subtypes.

The most salient feature of Fig. 1 is the linear relationship, on a 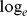-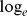 scale, between the gene-specific rates of evolution of H3N2 and H1N1 subtypes (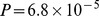; 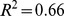) which indicates that the fast-evolving genes are the same in both subtypes. The simplest explanation, mechanistic in nature, would be that each gene accumulates substitutions at a gene-within-subtype specific rate, that is, follows a strict molecular clock [27]. However, this hypothesis is strongly rejected (Table S2 in File S1). An alternative explanation is that the fast-evolving genes (HA and NA) are expressed at the surface of the viral particle and are directly involved in the immune escape of the virus, while the slow-evolving genes all have internal functions [25]. NS2, which is also a fast-evolving protein, interacts directly with a host protein [28] and might therefore be involved in an 'arms race' with the host, leading up to high rates of evolution.

### Estimation of GC3 adjustment times

All the results above are consistent with previous reports, but they do not inform us on the time it takes for a virus to adjust to a new host. We define this duration by the period delimited by two events: a host change, followed by a change of viral GC3 content in the new host. Host changes were mapped using a simple maximum likelihood model [29], [30] on the phylogenetic trees estimated above. To ease computations, observed GC3 compositions were discretized (clustered) and, just like host changes, mapped on the estimated phylogenetic trees. This process was repeated for each gene of the influenza A genome, in each subtype.

Four remarks are necessary at this point. First, GC3 content is often used to monitor viral codon optimization after a host change, as in HIV-1 [31] and bacteriophages [32]. Furthermore, codon usage has been shown to be host-specific in the case of influenza viruses [16]. Here for instance, three human and swine data points in PB2 of H1N1 are in the avian GC3 cluster (Fig. S22 in File S1), and the phylogenetic analysis clearly demonstrates their recent avian origin (Fig. S1 in File S1). Yet, a change in GC3 composition does not necessarily reflect an adaptive process (see below). A critical asset of our computational approach is that we do not assume any adaptive process. Second, this process of GC3 change following cross-species transmission is obviously gradual. Similar processes have been documented both experimentally in HIV-1 [31] and computationally in bacteriophages [32], and no evidence ever suggested any form of stepwise (instantaneous) adjustment. Our discretization of the process can therefore be seen as a heuristic, but one that makes the computation more straightforward than fitting a diffusion process and determining the point at which *e.g.* 95% of the GC3 content has reached a new stationary phase. Third, an alternative to reconstructing changes of GC3 clusters would have been to reconstruct the sequences of ancestral genomes in order to compute GC3 contents on these ancestral genomes. However, while accuracy of ancestral sequence reconstruction can be high (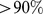) with four amino acid sequences [33], the actual performance of these methods with dozens of DNA sequences is unknown. Although ancestral state reconstruction might be more powerful, we opted here to reconstruct changes of GC3 contents directly. Fourth, phylogenetic uncertainty could be taken into account in our reconstructions of both host and GC3 changes, for instance by running the algorithm on all the trees sampled from the posterior distribution. We did not attempt to perform this computationally demanding analysis, as the objective here essentially aims at demonstrating the feasibility of the approach.

**Figure 2:**
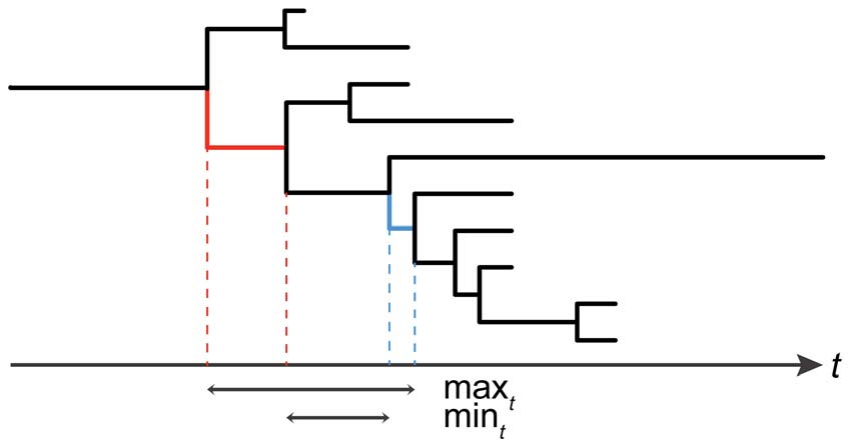
Estimation of adjustment times. Schematic representation of the method developed to estimate adjustment times. A host-switch event occurred along the red branch, and a GC3 cluster change occurred along the blue branch. Time 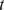 flows from the past to the present (bottom axis), and divergence times are estimated for nodes (see vertical broken lines). The two durations of interest are 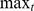 and 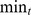. See text for details.

While we can estimate the dates beginning and terminating a branch on which each event (host-switch, GC3 cluster change) occurred, we do not know the exact time when each event took place. Nonetheless, we can define two durations, a maximum and a minimum duration indicated as 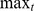 and 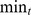, respectively, as in Fig. 2. The estimated adjustment periods used henceforth are the arithmetic averages of 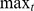 and 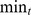.

### GC3 adjustment is faster in H3N2 than in H1N1

The GC3 adjustment process following a host change implicitly assumes that all three hosts have different GC3 compositions, and that the GC3 content of viruses tends to reflect that of their host. We detected a significant difference in the GC3 compositions of the transcriptome of all three hosts (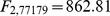, 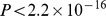), with birds having the largest GC3 content, followed by swine (Fig. S21 in File S1). Notably, GC3 contents of influenza viruses coming from specific hosts are ranked in the same order (Fig. S22-S23 in File S1), but tend to be twice as high as those of their host. This might explain why decreasing GC3 trends have been observed within host-specific influenza viruses ( *e.g.*, [16]). We observed such trends here, but most of them were not significant, even using robust regressions (Table S3 in File S1). This approximate stationarity of within-host GC3 contents gives further ground to our discretizing them. Indeed, our assumption of the existence of host-specific viral GC3 content demands that GC3 content be approximately constant in time. If this were not the case, we would not be able to draw horizontal lines in Fig. S22 and S23 (File S1) to represent boundaries between these host-specific GC3 contents.

**Figure 3:**
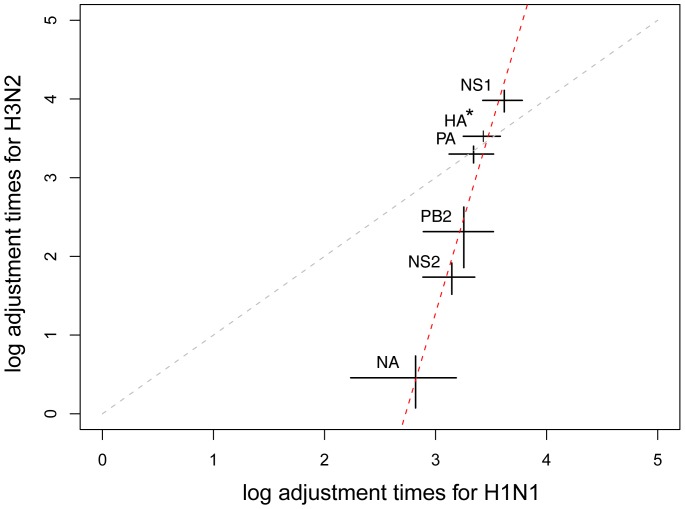
GC3 adjustment times of H3N2 *vs.* H1N1 viruses. Results are shown on a 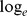-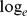 scale (in years). The gray line represents the first bisector (line of equation 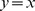), while the red line represents the linear fit to the data. Bars: SEMs (95% Highest Posterior Densities, not shown, tend to be larger – see Fig. S27 in File S1). 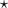: the HA value for H3N2 was tentatively derived using branches around the root node.

**Figure 4:**
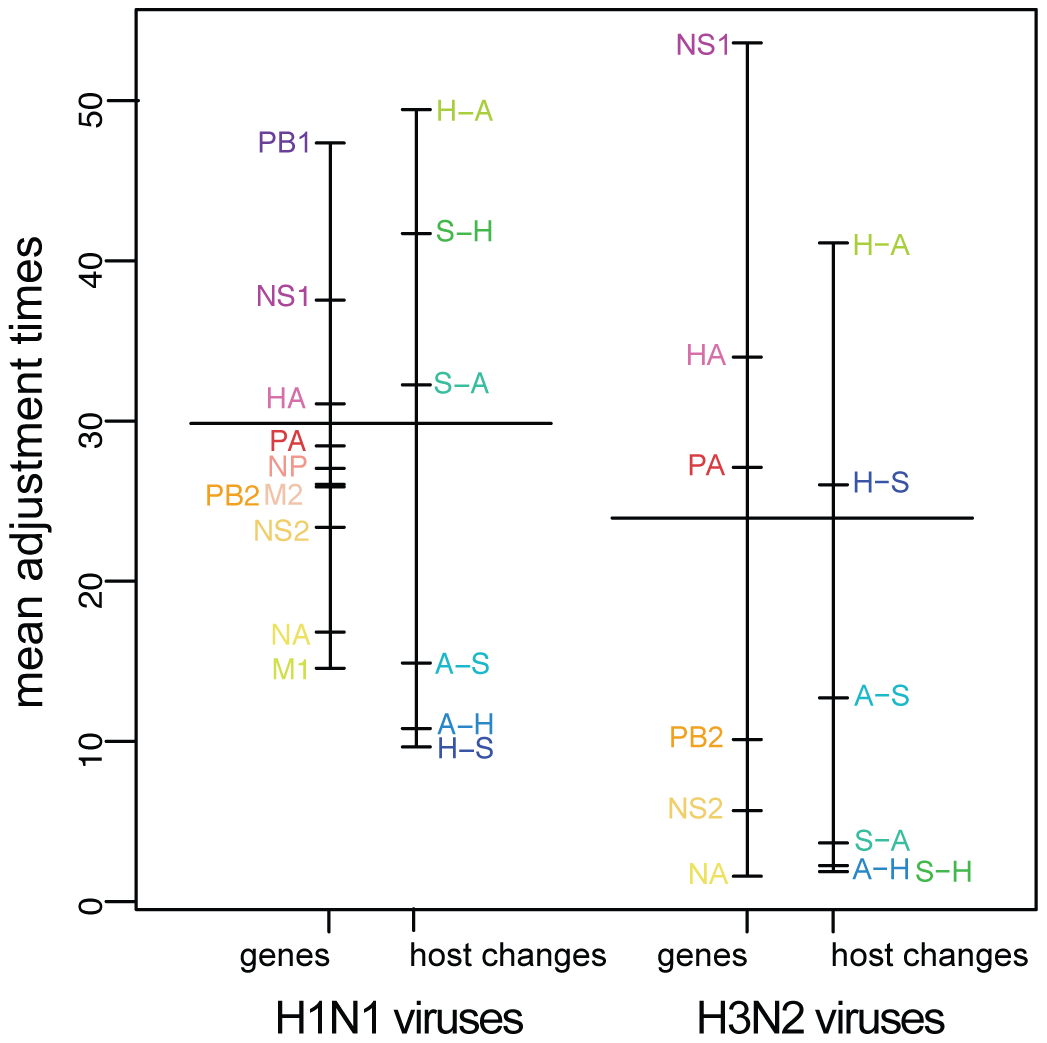
Factor effects in the linear model (ANOVA) that was fitted to adjustment times (in years). The directions of host change are avian-to-human (A-H), avian-to-swine (A-S), human-to-avian (H-A), human-to-swine (H-S), swine-to-avian (S-A) and swine-to-human (S-H). Adjustment times are in years. See text for details.

GC3 compositions of each gene of both H1N1 and H3N2 clustered into two groups (as determined by median split silhouettes; Fig. S22-S23, Table S3 in File S1) for most genes, typically clustering human and swine hosts together. In order to simplify the algorithm, we forced clustering to have two groups for each gene. As above for rates, we found a (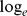-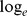) linear relationship in terms of GC3 adjustment durations between the two subtypes (Fig. 3). In particular, (i) GC3 content of H3N2 viruses adjusts faster than in H1N1 viruses (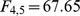, 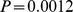) and (ii) the same ordering of genes exists for both subtypes (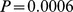; 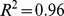). It is interesting to note that genes that are fast adjusting are also involved in the final stages of the viral cycle, NS2 mediating the export of newly synthesized RNPs from the nucleus [34] and NA mediating virus release from the infected cell [35]. Note that some genes are missing from Fig. 3 because they did not show any evidence for a combined host/GC3 change in our genome catchment. These are H3N2 genes PB1, NP, M2 and M1. Ordering for these genes in both subtypes was achieved by fitting a linear model (ANOVA) that describes mean GC3 change times as a function of two factors: gene segment and direction of host change. Results show that these two factors have a very significant effect (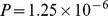 and 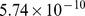, respectively; Fig. 4), so that three points can be made.

First, the rank ordering of genes by their adjustment time differs from their ranking in terms of rates of evolution (Fig. 1 *vs.* 3). While NA is the fastest adjusting gene, HA appears to be the second slowest adjusting gene when information around the root node is used (Methods), ranking just after NS1. While the position of NS1 is consistent with a previous study [14], that of HA is in contrast to its high rate of evolution and the body of literature implicating HA in host preference, or to the idea that HA and NA need to be co-evolving as they both target the same sialic acids on the host cells [5]. Here however, genes are not ordered with respect to their importance in evading ongoing host immune responses or other form of adaptation, but with respect to how their GC3 content adjusts to that of their host. One potential explanation of the difference between evolution and adjustment rates is that highly expressed influenza genes adjust rapidly since the virus highjacks the host translation machinery. However, while a number of studies have examined expression patterns of hosts genes [36], very little is known about expression patterns of viral genes during the course of an infection. Further research in viral transcriptomics is therefore warranted.

Second, some of the H1N1 genomes included in our alignment come from human viruses that reappeared in 1977 after a 20-year gap ( *e.g.*, [23]). The presence of these genomes in our data could potentially bias downwards our estimates of adjustment times for H1N1 viruses, or at least increase the variance of these time estimates [37]. However, none of the H1N1 viruses that reappeared in 1977 underwent a change of host, so that these viruses were not included in our calculation of adjustment times. More crucially, removing these genomes (in gray in Fig. S1-S10 in File S1) from the analyses estimating divergence times did not alter our estimates of 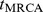, the age of the root (Fig. S24 in File S1). Furthermore, previous work showed that the rate of evolution of these reintroduced sequences is similar to that of seasonal H1N1 sequences [23]. Altogether, our results are therefore robust to the presence of these re-emergent viruses.

Third, GC3 adjustment times also depend on the direction of host change (Fig. 4). This little-studied aspect outside of human transmission [7] reveals that across both H1N1 and H3N2 subtypes, adjustment of human viruses to avian hosts is the slowest, while adjustment of viruses coming from an avian host is very fast, with an average 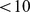 years (Fig. 4). On the other hand, subtypes show a difference in the GC3 adjustment speed of viruses coming from swine, with an average of 35 years for the H1N1 subtype *vs.* 5 years for H3N2. This difference between the adjustment dynamics of avian and swine viruses is somewhat unexpected, as swine is often considered to be the 'mixing vessel', harboring both types of sialic acids in its respiratory tracts and being therefore able to be infected by both avian and human viruses [38]. However, even if cross-species transmission requires some adaptive process, we show next that GC3 adjustment is probably not adaptive *per se*.

### GC3 adjustment reflects relaxed selective pressures

As viruses use the translational machinery of their host to translate their own mRNA, their codon usage and hence their GC3 content is expected to be under selective pressure to adapt to the pool of transfer RNA of their host [39]. To assess the role of selection during the GC3 content adjustment process, we tested for evidence of positive selection along the lineages starting from cross-species transmission and ending at the GC3 cluster change. Table S4 in File S1 shows that no such evidence could be detected. This result could be due to (i) the inclusion of 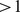 consecutive branches in the foreground lineages, (ii) selective forces acting on the background branches or to (iii) the non-distinction of the different directions of host change in this particular test. Current codon models allow us to have only one set of foreground branches [40], while with three hosts we would require six such sets, as done in the GC3 content analysis above (Fig. 4). It could also be possible to test for all possible combinations of foreground branches as recently proposed [41]. This procedure would circumvent the issue of using the same data twice, once to identify branches of interest and a second time to test for positive selection. However, while that approach would identify the branches along which positive selection can be detected [41], it would fail to test the specific hypothesis of presence of positive selection in the lineages between the cross-species transmission and the GC3 cluster change.

More critically, we find that shorter log adjustment times are significantly correlated with higher estimates of selection coefficients in the case of H3N2 (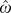 under 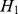; 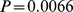, 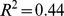), but not in the case of H1N1 viruses (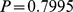). This result shows that relaxation of selective pressures plays a key role in the adjustment of H3N2 to a new host. While the lack of signal for H1N1 viruses might be due to 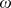 rate ratios that are specific to the direction of host change, the population genetics of the two subtypes might also explain the difference. While both H1N1 and H3N2 viruses are expected to undergo frequent bottlenecks during their spread among hosts populations (and hence increase drift), the larger population sizes found in H3N2, the dominant subtype for 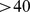 years since the 1968 pandemic, are expected to facilitate the action of selection. Altogether, the differential incidence between the two subtypes could explain the stronger role of relaxation of selective pressures in H3N2 across the three hosts studied here.

The origin of the adjustment process can be revealed by considering the effective number of codons (ENC). In spite of most relationships between ENC and GC3 being significantly positive (Fig. S25 in File S1), our data show no evidence for codon bias. Indeed, for the genes sampled here, ENC is never below the 35 threshold, which is usually taken as an indicator of strong codon bias [42] and ENC is almost always above 50 (Fig. S26-27 in File S1). Altogether, our results suggest that GC3 adjustment is essentially driven by mutational bias in H1N1 and H3N2 viruses, with a larger role of relaxed selective pressures in H3N2 viruses. Future work should focus on the differential dynamics of H1N1 and H3N2 subtypes, potentially taking inspiration from the use of nonhomogeneous models as in [23], but developed at the codon level.

## Conclusions

We showed here that studying cross-species transmissions of influenza A viruses that established themselves as stable lineages sheds some unsuspected light on the dynamics of two major subtypes. In particular, we demonstrated that both H1N1 and H3N2 subtypes have the same fast-adjusting genes in terms of GC3 content (Fig. 3), while H3N2 viruses adjust significantly faster (Fig. 3), in particular when coming from avian hosts (Fig. 4).

We also showed that two genes, NS2 and NA lead the pace of this adjustment process in both subtypes (Fig. 3). These genes play a key role in the final stages of the viral cycle in host cells (export of viral genome from nucleus and release of viral particles out of host cells, respectively), which consequently might be the limiting step of the adjustment process to a new host.

Although we did not attempt to validate the method on simulated data, extensions could consider using heterogeneous models [23]. Our results should also be validated by analyzing other, more extensive, data sets, beyond North America, to confirm (i) the relationship between adjustment rates of H1N1 and H3N2 viruses and (ii) the disconnect between viral adjustability and evolutionary rate. Finally, our results highlight the importance of obtaining complete genome data through surveillance program in order to unravel the dynamics of influenza viruses, and not just from the standpoint of GC3 adjustment. We argue that only such complete genome information will help us understand how emerging pathogens acquire the ability to be efficiently transmitted within their new host [43]. The most likely answer may not lie in the identification of signature amino acid sites, but rather in the determination of epistatic interaction of sites within [44] and among segments [45].

## Materials and Methods

### Data collection and alignment

Whole genome sequences of H1N1 and H3N2 subtypes of *all* influenza A viruses collected between 1900 and 2009 (as of January 2010) in North America (Mexico, the USA and Canada) in avian, human and swine hosts were retrieved from the Influenza Virus Resource [18]. Only one pandemic H1N1/2009 genome was included in this study, A/Canada-AB/RV1531/2009(H1N1) ; A/Saskatchewan/5131/2009(H1N1) is a seasonal (pre-pandemic) H1N1 virus [19]. The complete influenza genome includes the ten 'canonical' protein-coding genes [20], [21], consisting of the three polymerase subunits PB2, PB1 and PA, the hemagglutinin (HA) and neuraminidase (NA) antigens, the nucleoprotein (NP), ribonucleoprotein exporter (NS2, also called NEP), interferon antagonist (NS1), ion channel protein (M2) and the matrix protein (M1). Each gene was aligned at the protein level with Muscle [46] and back-translated to nucleotide alignments with Pal2Nal [47]. At this stage, manual adjustments were performed, in particular for the M2, M1, NS2 and NS1 genes. Improperly annotated or misaligned sequences were discarded. In total, our initial alignments contained 19,159 H1N1 and 10,498 H3N2 genes (Table S1 in File S1).

### Sequence clustering

In order to decrease sample size to make alignments amenable to phylogenetic analysis without compromising data quality, sequences similar at the 99% threshold were removed from the alignment as done in a previous study [21]. Briefly, pairwise genetic distances were computed with PAUP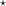 [48] under the GTR + 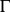 + I model of evolution. Sequences were then clustered with DOTUR [49] at the 99% similarity level using the nearest neighbor algorithm. We checked that each cluster thus identified contained sequences coming from only one single host (Fig. S28 in File S1); when this was not the case, a sequence from the most common host was selected at random; we then tested that such cases correspond to unsustained cross-species transmission events (Fig. S1-S20 in File S1), so that these cases are not included in our dating analyses. Note that the H1N1 1918 human virus [50] was not included in the final data. Accession numbers of the genes retained are shown in Fig. S1-S20 in File S1.

### Phylogenetic analyses

The most appropriate model of evolution for each of the ten 'canonical' gene of each subtype was chosen according to the Akaike Information Criterion in jModelTest [51] (Table S5 in File S1). The strict molecular clock was tested with PAML ver. 4.4b [52] under the TipDate model [53] using the trees estimated under a relaxed molecular clock implemented in BEAST ver. 1.6.1 [54].

Divergence times were estimated by assuming an uncorrelated lognormal prior distribution to describe the evolution of the rates of evolution [22]. A Bayesian coalescent skyline prior with ten breakpoints and stepwise splines [55] was placed on times. Markov chain Monte Carlo samplers were run for 1 billion steps with a thinning of 5000 steps for each gene, and in duplicate to check for convergence. Tracer (tree.bio.ed.ac.uk/software) was used to monitor the runs and to determine the burn-in periods. An in-house Perl script was then used to remove the burn-in period of each pair of runs, concatenate the log files and run TreeAnnotator [54]. The relaxed-clock trees are, by construction, rooted ( *e.g.*, [21], [56]).

### Timing GC3 adjustment after a host change

Host changes were determined by mapping ancestral hosts on the phylogeny of each gene under a simple maximum likelihood approach [29], [30] assuming that all three hosts had the same rate of change (more sophisticated models where all rates were different tended to exhibit convergence issues on our data). The APE library [57] in R [58] was used for this purpose. Placement of host-switch events was determined manually according to reconstructed ancestral mapping (Tables S6-S7 in File S1).

GC3 content and effective number of codons (ENC) were calculated for each gene with GCUA [59]. Gene-specific GC3 distributions were discretized by Partition Around Medoids clustering, where the optimal number of clusters was determined by Median Split Silhouettes (for details, see [60]). Ancestral GC3 cluster assignments were reconstructed with a maximum likelihood model as above [29], [30]. Stabilization of GC3 content was inferred when (i) a host change occurred along a lineage and (ii) a subsequent change of GC3 cluster occurred. Because of the uncertain ancestral reconstructions for the two branches emanating from the root, these two branches were left out of the computations. Adjustment times were inferred as depicted in Fig. 2.

We also downloaded from *ensembl* release 62 [61], available at ensembl.org/info/data/ftp, the complete transcriptomes of the hosts: chicken ( *Gallus gallus* – chosen arbitrarily out of the three completed bird genomes with turkey and zebra finch, as of October 2011), human ( *Homo sapiens*) and pig ( *Sus scrofa*). The transcriptomes were analyzed with GCUA and tested for transcriptomes-wide differences in their GC3 composition. Genes with no termination signal as *per* GCUA or with 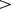
20,000 bases were discarded, leaving 17,087 avian genes, 46,040 human genes and 14,056 swine genes.

### Detection of selection

In order to test for positive selection at some sites along the branches between a host change and a change of GC3 cluster, we ran branch-site codon models [40] as implemented in codeml ver. 4.4d [52]. Nonsynonymous to synonymous rate ratios (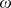) are used to measure selection in protein-coding genes, with 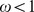 indicating negative selection, 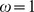 neutral evolution and 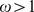 positive selection. Branch-site codon models allow 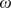 to vary both along the sequence and along some pre-specified branches, called the foreground branches, while the ratio in the other branches, or background branches, is kept constant and 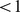. A likelihood ratio test (LRT) was used to test the null hypothesis 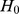 that there is no positive selection at any site along the foreground branches. The alternative 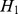 is that there is evidence for positive selection at some sites in the foreground branches. The LRT test statistic was conservatively assumed to follow a 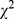 distribution with one degree of freedom rather than the appropriate mixture distribution [40]. Sites potentially evolving adaptively were inferred with a Bayes empirical Bayes method [62] at the 95% posterior probability cutoff. All regressions performed in this study were based on robust linear models [63].

## Supporting Information

File S1 **This file contains the supplementary tables S1-S7 and supplementary figures S1-S28.**
(PDF)
