# Supplementary material for: Phylodynamics of the Emergence of Influenza Viruses after Cross-Species Transmission
Source: PLoS One. 2013 Dec 16;8(12):e82486. doi: 10.1371/journal.pone.0082486 (PMC3865002; doi:10.1371/journal.pone.0082486)
Supplement: File S1 — This file contains the supplementary tables S1-S7 and supplementary figures S1-S28. (PDF) [file pone.0082486.s001.pdf]

Table S1: **Genomic data used in this study.** $sl$: sequence length (nucleotides); $ns_{\mathrm{before-clust}}$: number of sequences before clustering; $ns_{\mathrm{after-clust}}$: number of sequences after clustering with `DOTUR`.

| Subtype | Gene | $sl$ | $ns_{\mathrm{before-clust}}$ | $ns_{\mathrm{after-clust}}$ |
|---|---|---|---|---|
| H1N1 | PB2 | 2277 | 1900 | 81 |
| | PB1 | 2271 | 1932 | 82 |
| | PA | 2148 | 1931 | 75 |
| | HA | 1698 | 1921 | 87 |
| | NP | 1494 | 1923 | 77 |
| | NA | 1407 | 1910 | 79 |
| | M2 | 291 | 1906 | 59 |
| | M1 | 756 | 1906 | 66 |
| | NS2 | 363 | 1915 | 66 |
| | NS1 | 657 | 1915 | 80 |
| H3N2 | PB2 | 2277 | 1107 | 45 |
| | PB1 | 2271 | 1110 | 45 |
| | PA | 2148 | 1088 | 36 |
| | HA | 1698 | 1106 | 59 |
| | NP | 1494 | 1064 | 39 |
| | NA | 1407 | 1015 | 55 |
| | M2 | 291 | 907 | 32 |
| | M1 | 756 | 907 | 29 |
| | NS2 | 363 | 1104 | 48 |
| | NS1 | 657 | 1090 | 38 |

Table S2: **Tests of the molecular clock assumption**. $\ell_{\text{noclock}}$: log-likelihood without the clock assumption; $\ell_{\text{clock}}$: log-likelihood under the clock assumption; $ns$: number of sequences; $X^2$: test statistic (twice the log-likelihood difference); df: degree of freedom; $P$: $P$-value.

| Subtype | Gene | $\ell_{\text{noclock}}$ | $\ell_{\text{clock}}$ | $ns$ | $X^2$ | df | $P$ |
|---|---|---|---|---|---|---|---|
| H1N1 | PB2 | -21268.37 | -21497.97 | 81 | 459.21 | 79 | $5.604 \times 10^{-55}$ |
| | PB1 | -21193.70 | -21385.71 | 82 | 384.03 | 80 | $2.801 \times 10^{-41}$ |
| | PA | -19708.20 | -19957.45 | 75 | 498.50 | 73 | $1.282 \times 10^{-64}$ |
| | HA | -17590.60 | -17828.70 | 87 | 476.21 | 85 | $9.481 \times 10^{-56}$ |
| | NP | -12609.96 | -12761.42 | 77 | 302.91 | 75 | $3.708 \times 10^{-29}$ |
| | NA | -12671.49 | -12814.82 | 79 | 286.65 | 77 | $6.612 \times 10^{-26}$ |
| | M2 | -1681.90 | -1727.97 | 59 | 92.14 | 57 | 0.0022 |
| | M1 | -4855.03 | -4909.53 | 66 | 109.01 | 64 | 0.0004 |
| | NS2 | -2585.38 | -2721.16 | 66 | 271.56 | 64 | $2.221 \times 10^{-27}$ |
| | NS1 | -6115.67 | -6217.65 | 80 | 203.95 | 78 | $3.277 \times 10^{-13}$ |
| H3N2 | PB2 | -13255.71 | -13331.62 | 45 | 151.81 | 43 | $4.673 \times 10^{-14}$ |
| | PB1 | -11495.45 | -11558.25 | 45 | 125.59 | 43 | $5.107 \times 10^{-10}$ |
| | PA | -10460.50 | -10518.00 | 36 | 116.99 | 34 | $4.844 \times 10^{-11}$ |
| | HA | -10919.79 | -11000.53 | 59 | 161.46 | 57 | $6.317 \times 10^{-12}$ |
| | NP | -7729.46 | -7789.52 | 39 | 120.11 | 37 | $1.032 \times 10^{-10}$ |
| | NA | -8360.71 | -8429.88 | 55 | 138.34 | 53 | $1.501 \times 10^{-09}$ |
| | M2 | -1254.13 | -1290.31 | 32 | 72.35 | 30 | $2.339 \times 10^{-05}$ |
| | M1 | -2805.23 | -2826.02 | 29 | 41.58 | 27 | 0.0362 |
| | NS2 | -2006.66 | -2155.28 | 48 | 297.24 | 46 | $1.816 \times 10^{-38}$ |
| | NS1 | -3478.96 | -3519.68 | 37 | 81.44 | 35 | $1.443 \times 10^{-05}$ |

Table S3: **Significance of the trend observed in GC3 clusters.** $n_{MSS}$ is the number of clusters estimated by median split silhouettes. $P$-values are derived from robust linear regressions. Significant results ($\alpha < 0.01$) are in bold. Cluster identifiers are arbitrary.

| Subtype | Gene | $n_{MSS}$ | GC3 cluster 1 | GC3 cluster 2 |
|---|---|---|---|---|
| H1N1 | PB2 | 2 | **0.0002** | $\mathbf{9.508}\times10^{-8}$ |
| | PB1 | 2 | 0.7029 | 0.3913 |
| | PA | 2 | 0.1839 | 0.5605 |
| | HA | 3 | 0.0115 | 0.6235 |
| | NP | 2 | 0.4107 | **0.0001** |
| | NA | 2 | 0.8631 | **0.0079** |
| | M2 | 9 | 0.1014 | 0.2161 |
| | M1 | 9 | 0.9716 | 0.8673 |
| | NS2 | 2 | 0.7784 | 0.4993 |
| | NS1 | 2 | 0.0666 | 0.9948 |
| H3N2 | PB2 | 2 | **0.0002** | **0.0056** |
| | PB1 | 2 | 0.3329 | 0.9434 |
| | PA | 2 | 0.2945 | 0.0762 |
| | HA | 2 | 0.4907 | $\mathbf{4.828}\times10^{-5}$ |
| | NP | 2 | 0.8473 | 0.1514 |
| | NA | 2 | 0.2762 | 0.4825 |
| | M2 | 9 | 0.0238 | 0.1936 |
| | M1 | 2 | 0.6171 | 0.8301 |
| | NS2 | 2 | 0.8538 | 0.0274 |
| | NS1 | 7 | 0.5675 | 0.8012 |

Table S4: **Branch-site test of positive selection during adjustment to a new host**. Hyp: hypothesis; $\ell$: log-likelihood; $np$: number of parameters; $P$: $P$-value; $\hat{\omega}$: estimated $\omega$ value; $\hat{p}_{\omega}$: proportion of sites (with $\hat{\omega} < 1$ under $H_0$, or $\hat{\omega} > 1$ under $H_1$); na: not applicable; long dash (—): data not available.

| Subtype | Gene | Hyp | $\ell$ | $np$ | $P$ | $\hat{\omega}$ | $\hat{p}_{\omega}$ | Sites |
|---|---|---|---|---|---|---|---|---|
| H1N1 | PB2 | $H_0$ | -20430.45 | 164 | na | 0.03 | 0.97 | na |
| | | $H_1$ | -20430.45 | 165 | 1.0000 | 1.00 | 0.00 | none |
| | PB1 | $H_0$ | -20277.40 | 166 | na | 0.03 | 0.96 | na |
| | | $H_1$ | -20277.40 | 167 | 0.9980 | 1.00 | 0.03 | 75 E, 327 R, 741 A |
| | PA | $H_0$ | -19132.36 | 152 | na | 0.03 | 0.96 | na |
| | | $H_1$ | -19132.36 | 153 | 0.9917 | 1.00 | 0.00 | none |
| | HA | $H_0$ | -17467.62 | 176 | na | 0.06 | 0.85 | na |
| | | $H_1$ | -17467.32 | 177 | 0.4409 | 2.08 | 0.00 | 277 G |
| | NP | $H_0$ | -12072.00 | 156 | na | 0.03 | 0.93 | na |
| | | $H_1$ | -12072.00 | 157 | 1.0000 | 1.00 | 0.03 | none |
| | NA | $H_0$ | -12965.21 | 160 | na | 0.06 | 0.82 | na |
| | | $H_1$ | -12965.21 | 161 | 0.9952 | 1.00 | 0.00 | none |
| | M2 | $H_0$ | -1733.00 | 120 | na | 0.07 | 0.53 | na |
| | | $H_1$ | -1733.00 | 121 | 1.0000 | 1.00 | 0.00 | none |
| | M1 | $H_0$ | -4747.81 | 134 | na | 0.02 | 0.97 | na |
| | | $H_1$ | -4747.81 | 135 | 1.0000 | 1.00 | 0.01 | none |
| | NS2 | $H_0$ | -2538.76 | 134 | na | 0.04 | 0.80 | na |
| | | $H_1$ | -2538.61 | 135 | 0.5795 | 1.56 | 0.02 | none |
| | NS1 | $H_0$ | -6083.67 | 162 | na | 0.12 | 0.83 | na |
| | | $H_1$ | -6083.67 | 163 | 1.0000 | 1.00 | 0.00 | none |
| H3N2 | PB2 | $H_0$ | -12423.36 | 92 | na | 0.03 | 0.95 | na |
| | | $H_1$ | -12423.21 | 93 | 0.5828 | 1.32 | 0.02 | 65 E |
| | PB1 | $H_0$ | — | — | na | — | — | na |
| | | $H_1$ | — | — | — | — | — | na |
| | PA | $H_0$ | -9760.82 | 74 | na | 0.03 | 0.96 | na |
| | | $H_1$ | -9760.82 | 75 | 1.0000 | 1.00 | 0.02 | 388 S |
| | HA | $H_0$ | — | — | na | — | — | na |
| | | $H_1$ | — | — | — | — | — | na |
| | NP | $H_0$ | — | — | na | — | — | na |
| | | $H_1$ | — | — | — | — | — | na |
| | NA | $H_0$ | -8058.08 | 112 | na | 0.05 | 0.66 | na |
| | | $H_1$ | -8058.06 | 113 | 0.8468 | 1.63 | 0.09 | none |
| | M2 | $H_0$ | — | — | na | — | — | na |
| | | $H_1$ | — | — | — | — | — | na |
| | M1 | $H_0$ | — | — | na | — | — | na |
| | | $H_1$ | — | — | — | — | — | na |
| | NS2 | $H_0$ | -1936.06 | 98 | na | 0.05 | 0.81 | na |
| | | $H_1$ | -1935.31 | 99 | 0.2203 | 2.22 | 0.04 | none |
| | NS1 | $H_0$ | -3412.36 | 76 | na | 0.09 | 0.72 | na |
| | | $H_1$ | -3412.36 | 77 | 1.0000 | 1.00 | 0.00 | none |

Table S5: **Best-fit substitution models of evolution.** Models were selected based on the Akaike Information Criterion (AIC).

| Subtype | Gene | AIC-selected model | Closest model in BEAST |
|---|---|---|---|
| H1N1 | PB2 | GTR + $\Gamma_4$ + I | GTR + $\Gamma_4$ + I |
| | PB1 | GTR+ $\Gamma_4$ | GTR + $\Gamma_4$ |
| | PA | GTR + $\Gamma_4$ + I | GTR + $\Gamma_4$ + I |
| | HA | GTR + $\Gamma_4$ + I | GTR + $\Gamma_4$ + I |
| | NP | GTR + $\Gamma_4$ + I | GTR + $\Gamma_4$ + I |
| | NA | GTR + $\Gamma_4$ | GTR + $\Gamma_4$ |
| | M2 | TPM2uf + $\Gamma_4$ | HKY+ $\Gamma_4$ |
| | M1 | TVM + $\Gamma_4$ + I | GTR + $\Gamma_4$ + I |
| | NS2 | TPM1uf + $\Gamma_4$ | HKY+ $\Gamma_4$ |
| | NS1 | TVM + $\Gamma_4$ + I | GTR + $\Gamma_4$ + I |
| H3N2 | PB2 | GTR + $\Gamma_4$ + I | GTR + $\Gamma_4$ + I |
| | PB1 | GTR+ $\Gamma_4$ | GTR + $\Gamma_4$ |
| | PA | TVM + $\Gamma_4$ | GTR + $\Gamma_4$ |
| | HA | GTR + I | GTR + I |
| | NP | TVM + $\Gamma_4$ | GTR + $\Gamma_4$ |
| | NA | GTR + I | GTR + I |
| | M2 | HKY + I | HKY + I |
| | M1 | GTR + I | GTR + I |
| | NS2 | TVM + I | HKY + I |
| | NS1 | TVM + I | GTR + $\Gamma_4$ + I |

Table S6: **Timing of inter-species influenza transmission events of H1N1 viruses.** The directions of host-switch (SwitchDir) are avian-to-human (A-H), avian-to-swine (A-S), human-to-avian (H-A), human-to-swine (H-S), swine-to-avian (S-A) and swine-to-human (S-H). Lower bound $\mathcal{L}$, upper bound $\mathcal{U}$ and mean($\mathcal{L}$,$\mathcal{U}$) times derived from figures S28-S10 are in years; na: not applicable. See text for details.

| Gene | SwitchDir | $\mathcal{L}$ | $\mathcal{U}$ | mean($\mathcal{L}$,$\mathcal{U}$) |
|---|---|---|---|---|
| PB2 | H-A | 0.0000 | 59.0541 | 29.52705 |
| PB2 | H-A | 17.8708 | 79.7203 | 48.79555 |
| PB2 | H-A | 26.8718 | 88.8288 | 57.8503 |
| PB2 | H-A | 29.0272 | 90.9842 | 60.0057 |
| PB2 | S-A | 0.0000 | 13.8794 | 6.9397 |
| PB2 | S-H | 0.0000 | 13.724 | 6.862 |
| PB2 | A-S | 0.0000 | 19.7952 | 9.8976 |
| PB2 | A-S | 2.1554 | 21.7952 | 11.9753 |
| PB2 | S-H | 0.0000 | 2.4756 | 1.2378 |
| PB1 | S-H | 65.1659 | 90.2625 | 77.7142 |
| PB1 | S-H | 65.1659 | 95.0147 | 80.0903 |
| PB1 | S-H | 67.2448 | 93.0296 | 80.1372 |
| PB1 | S-H | 76.3068 | 100.1435 | 88.22515 |
| PB1 | S-H | 67.2448 | 100.0146 | 83.6297 |
| PB1 | S-H | 76.3068 | 107.0146 | 91.6607 |
| PB1 | S-H | 0.0000 | 4.5933 | 2.29665 |
| PB1 | A-H | 0.0000 | 11.3698 | 5.6849 |
| PB1 | A-S | 0.0000 | 29.7703 | 14.88515 |
| PB1 | A-S | 8.028 | 41.123 | 24.5755 |
| PB1 | A-S | 26.4285 | 41.123 | 33.77575 |
| PB1 | H-S | 8.028 | 29.7532 | 18.8906 |
| PB1 | H-S | 0.0000 | 18.4005 | 9.20025 |
| PA | A-H | 0.0000 | 0.5153 | 0.25765 |
| PA | H-S | 0.0000 | 2.9156 | 1.4578 |
| PA | H-S | 0.0000 | 2.9927 | 1.49635 |
| PA | S-H | 0.0000 | 19.7675 | 9.88375 |
| PA | S-H | 4.4921 | 27.1778 | 15.83495 |
| PA | S-H | 13.9118 | 62.1778 | 38.0448 |
| PA | S-H | 57.0224 | 79.1779 | 68.10015 |
| PA | S-A | 0.0000 | 48.8197 | 24.40985 |
| PA | S-A | 0.0000 | 49.9711 | 24.98555 |
| PA | S-A | 0.0000 | 50.9105 | 25.45525 |
| PA | S-A | 0.0000 | 52.236 | 26.118 |
| PA | S-A | 45.2189 | 69.6726 | 57.44575 |
| PA | S-A | 51.1326 | 72.4583 | 61.79545 |
| PA | S-A | 18.8265 | 39.5047 | 29.1656 |
| PA | S-A | 20.9647 | 58.4414 | 39.70305 |
| HA | S-H | 9.775 | 11.1698 | 10.4724 |
| HA | S-H | 13.0742 | 17.1778 | 15.126 |
| HA | S-H | 16.2341 | 23.3098 | 19.77195 |
| HA | S-H | 22.3661 | 28.4685 | 25.4173 |
| HA | S-H | 27.5248 | 36.0261 | 31.77545 |
| HA | S-H | 13.0742 | 36.0261 | 24.55015 |
| HA | S-A | 0.0000 | 6.4411 | 3.22055 |
| HA | S-A | 0.0000 | 24.3768 | 12.1884 |
| HA | S-A | 0.0000 | 17.7318 | 8.8659 |
| HA | S-A | 0.0000 | 12.5731 | 6.28655 |

| Gene | SwitchDir | $\mathcal{L}$ | $\mathcal{U}$ | mean($\mathcal{L}$,$\mathcal{U}$) |
|---|---|---|---|---|
| HA | A-H | 0.0000 | 6.132 | 3.066 |
| HA | H-S | 0.0000 | 5.1587 | 2.57935 |
| HA | S-H | 0.0000 | 6.645 | 3.3225 |
| HA | A-H | 0.0000 | 17.9357 | 8.96785 |
| HA | A-H | 0.0000 | 11.2907 | 5.64535 |
| HA | H-S | 0.0000 | 11.7737 | 5.88685 |
| HA | S-A | 5.116 | 62.1144 | 33.6152 |
| HA | S-A | 5.116 | 75.0718 | 40.0939 |
| HA | S-A | 9.7729 | 75.0718 | 42.42235 |
| HA | S-A | 4.0793 | 58.9798 | 31.52955 |
| HA | S-H | 0.0000 | 27.0984 | 13.5492 |
| HA | S-H | 4.1718 | 35.9054 | 20.0386 |
| HA | S-H | 10.3767 | 45.1565 | 27.7666 |
| HA | S-H | 10.3767 | 75.6431 | 43.0099 |
| HA | S-H | 18.0581 | 75.6431 | 46.8506 |
| HA | S-H | 52.088 | 81.9053 | 66.99665 |
| HA | S-H | 66.2077 | 95.8964 | 81.05205 |
| HA | S-H | 68.8032 | 98.4919 | 83.64755 |
| HA | S-H | 71.3987 | 99.7425 | 85.5706 |
| HA | S-H | 72.6493 | 106.9053 | 89.7773 |
| HA | S-H | 75.8121 | 106.9106 | 91.36135 |
| HA | H-S | 0.0000 | 7.1628 | 3.5814 |
| NP | S-A | 0.0000 | 3.9924 | 1.9962 |
| NP | S-H | 0.0000 | 24.9926 | 12.4963 |
| NP | S-H | 11.8795 | 33.6349 | 22.7572 |
| NP | S-H | 15.5536 | 35.0531 | 25.30335 |
| NP | S-H | 15.6977 | 35.2052 | 25.45145 |
| NP | S-H | 15.8498 | 41.6349 | 28.74235 |
| NP | S-H | 81.2717 | 101.5028 | 91.38725 |
| NP | S-H | 82.1474 | 104.6349 | 93.39115 |
| NP | A-S | 9.1608 | 14.4679 | 11.81435 |
| NP | S-A | 9.1608 | 16.61 | 12.8854 |
| NP | A-S | 9.1608 | 19.287 | 14.2239 |
| NP | S-A | 9.1608 | 19.9924 | 14.5766 |
| NP | S-H | 0.0000 | 17.7596 | 8.8798 |
| NP | S-H | 0.0000 | 24.9926 | 12.4963 |
| NA | S-A | 0.0000 | 1.6493 | 0.82465 |
| NA | S-A | 0.0000 | 4.7018 | 2.3509 |
| NA | S-A | 2.3596 | 9.703 | 6.0313 |
| NA | S-A | 21.8701 | 55.4591 | 38.6646 |
| NA | S-A | 25.9226 | 55.4591 | 40.69085 |
| NA | S-A | 0.0000 | 24.163 | 12.0815 |
| M2 | S-A | 0.0000 | 48.8792 | 24.4396 |
| M2 | S-A | 3.8246 | 50.2708 | 27.0477 |
| M1 | S-A | 19.5323 | 54.4618 | 36.99705 |
| M1 | A-H | 0.0000 | 5.4589 | 2.72945 |
| M1 | A-H | 10.8406 | 16.4915 | 13.66605 |
| M1 | A-H | 13.7748 | 27.0726 | 20.4237 |
| M1 | H-S | 0.0000 | 10.5811 | 5.29055 |
| M1 | S-A | 0.0000 | 7.4507 | 3.72535 |
| M1 | A-H | 10.8406 | 27.0726 | 18.9566 |

| Gene | SwitchDir | $\mathcal{L}$ | $\mathcal{U}$ | mean($\mathcal{L}$,$\mathcal{U}$) |
|---|---|---|---|---|
| NS2 | S-A | 30.3848 | 76.7627 | 53.57375 |
| NS2 | S-A | 50.8615 | 98.7648 | 74.81315 |
| NS2 | S-A | 50.7541 | 98.7648 | 74.75945 |
| NS2 | S-A | 37.6968 | 74.7648 | 56.2308 |
| NS2 | S-A | 50.1823 | 97.7648 | 73.97355 |
| NS2 | S-H | 0.0000 | 3.2697 | 1.63485 |
| NS2 | H-S | 0.0000 | 6.7476 | 3.3738 |
| NS2 | H-S | 2.8481 | 9.1394 | 5.99375 |
| NS2 | H-S | 3.5777 | 15.6019 | 9.5898 |
| NS2 | A-S | 0.0000 | 3.6462 | 1.8231 |
| NS2 | S-H | 0.0000 | 4.4575 | 2.22875 |
| NS2 | S-H | 0.0000 | 7.6758 | 3.8379 |
| NS2 | H-S | 0.0000 | 1.8375 | 0.91875 |
| NS2 | S-H | 0.0000 | 5.7133 | 2.85665 |
| NS2 | H-S | 12.8048 | 24.4011 | 18.60295 |
| NS2 | H-S | 26.0879 | 31.8459 | 28.9669 |
| NS2 | H-S | 28.5053 | 38.1391 | 33.3222 |
| NS2 | A-S | 5.581 | 16.903 | 11.242 |
| NS2 | A-S | 18.8641 | 24.3478 | 21.60595 |
| NS2 | A-S | 21.2815 | 30.641 | 25.96125 |
| NS2 | S-H | 5.6073 | 15.1206 | 10.36395 |
| NS2 | S-H | 8.0247 | 21.4138 | 14.71925 |
| NS2 | S-H | 0.5799 | 8.1307 | 4.3553 |
| NS1 | S-H | 13.1126 | 61.2579 | 37.18525 |
| NS1 | S-H | 56.0008 | 67.93 | 61.9654 |
| NS1 | S-H | 76.5844 | 90.0381 | 83.31125 |
| NS1 | S-H | 80.4821 | 94.9298 | 87.70595 |
| NS1 | S-H | 81.4489 | 96.9298 | 89.18935 |
| NS1 | S-H | 81.8215 | 93.9298 | 87.87565 |
| NS1 | S-A | 0.0000 | 64.9299 | 32.46495 |
| NS1 | S-A | 15.4339 | 68.9299 | 42.1819 |
| NS1 | S-A | 29.0852 | 75.9298 | 52.5075 |
| NS1 | S-A | 29.0852 | 93.9299 | 61.50755 |
| NS1 | S-A | 35.5561 | 90.9299 | 63.243 |
| NS1 | A-S | 0.0000 | 20.3685 | 10.18425 |
| NS1 | A-S | 0.9022 | 19.3685 | 10.13535 |
| NS1 | A-S | 0.7203 | 18.5105 | 9.6154 |
| NS1 | H-S | 1.9464 | 15.1431 | 8.54475 |
| NS1 | A-S | 1.9464 | 20.5105 | 11.22845 |
| NS1 | H-S | 0.7203 | 13.1431 | 6.9317 |
| NS1 | S-H | 0.0000 | 6.1245 | 3.06225 |
| NS1 | A-H | 12.5741 | 20.5105 | 16.5423 |
| NS1 | A-H | 13.0962 | 20.5105 | 16.8033 |
| NS1 | A-H | 7.7925 | 18.5105 | 13.1515 |
| NS1 | A-H | 9.0186 | 20.5105 | 14.76455 |

Table S7: **Timing of inter-species influenza transmission events of H3N2 viruses.** The directions of host-switch (SwitchDir) are avian-to-human (A-H), avian-to-swine (A-S), human-to-avian (H-A), human-to-swine (H-S), swine-to-avian (S-A) and swine-to-human (S-H). Lower bound $\mathcal{L}$, upper bound $\mathcal{U}$ and mean($\mathcal{L}$,$\mathcal{U}$) times derived from figures S11-S20 are in years; na: not applicable. See text for details.

| Gene | SwitchDir | $\mathcal{L}$ | $\mathcal{U}$ | mean($\mathcal{L}$,$\mathcal{U}$) |
|---|---|---|---|---|
| PB2 | A-S | 4.4218 | 14.3549 | 9.38835 |
| PB2 | A-S | 9.2915 | 24.7574 | 17.02445 |
| PB2 | A-S | 13.2389 | 26.7574 | 19.99815 |
| PB2 | A-H | 0.0000 | 3.8998 | 1.9499 |
| PB2 | S-A | 0.0000 | 4.4243 | 2.21215 |
| PB1 | na | na | na | na |
| PA | H-A | 10.7789 | 34.645 | 22.71195 |
| PA | H-A | 17.9375 | 44.645 | 31.29125 |
| PA | H-A | 30.6808 | 54.645 | 42.6629 |
| PA | H-A | 30.6808 | 55.645 | 43.1629 |
| PA | H-A | 33.1834 | 63.6449 | 48.41415 |
| PA | A-S | 0.7118 | 38.7666 | 19.7392 |
| PA | A-S | 0.7118 | 42.7666 | 21.7392 |
| PA | H-S | 0.7118 | 59.645 | 30.1784 |
| PA | H-S | 0.7118 | 63.645 | 32.1784 |
| PA | S-A | 0.0000 | 11.1514 | 5.5757 |
| PA | A-S | 6.167 | 43.7665 | 24.96675 |
| PA | H-S | 0.0000 | 6.6961 | 3.34805 |
| PA | H-S | 6.167 | 64.6449 | 35.40595 |
| PA | A-S | 6.5804 | 39.7665 | 23.17345 |
| PA | H- S | 6.5804 | 60.6449 | 33.61265 |
| PA | S-A | 0.0000 | 2.2827 | 1.14135 |
| PA | A-S | 7.185 | 42.7666 | 24.9758 |
| PA | H-S | 7.185 | 63.645 | 35.415 |
| PA | A-S | 7.185 | 44.7666 | 25.9758 |
| PA | H-S | 7.185 | 65.645 | 36.415 |
| HA | H-A | 23.9784 | 44.0007 | 33.98955 |
| NP | na | na | na | na |
| NA | H-A | 0.0000 | 1.1322 | 0.5661 |
| NA | A-S | 0.0000 | 0.2998 | 0.1499 |
| NA | H-A | 0.0582 | 1.4902 | 0.7742 |
| NA | H-A | 0.358 | 5.5838 | 2.9709 |
| NA | H-A | 0.0582 | 5.5838 | 2.821 |
| NA | A-S | 0.0000 | 4.3934 | 2.1967 |
| M2 | na | na | na | na |
| M1 | na | na | na | na |
| NS2 | H-A | 0.0000 | 15.2581 | 7.62905 |
| NS2 | H-A | 0.0000 | 28.2581 | 14.12905 |
| NS2 | A-S | 0.0000 | 3.3667 | 1.68335 |
| NS2 | S-A | 0.0000 | 4.1403 | 2.07015 |
| NS2 | H-S | 0.0000 | 3.2146 | 1.6073 |
| NS2 | A-S | 0.0000 | 7.507 | 3.7535 |
| NS2 | S-A | 0.9318 | 6.0113 | 3.47155 |
| NS2 | A-S | 5.0721 | 9.378 | 7.22505 |
| NS2 | A-S | 7.9188 | 14.5001 | 11.20945 |
| NS2 | S-A | 3.7785 | 11.1334 | 7.45595 |

| Gene | SwitchDir | $\mathcal{L}$ | $\mathcal{U}$ | mean($\mathcal{L}$,$\mathcal{U}$) |
|---|---|---|---|---|
| NS2 | A-S | 7.9188 | 14.5001 | 11.20945 |
| NS2 | A-H | 0.0000 | 5.1221 | 2.56105 |
| NS2 | A-S | 1.2761 | 5.1221 | 3.1991 |
| NS2 | S-H | 0.0000 | 4.4907 | 2.24535 |
| NS1 | H-A | 0.0000 | 55.7515 | 27.87575 |
| NS1 | H-A | 43.6675 | 69.5093 | 56.5884 |
| NS1 | H-A | 43.6675 | 73.9359 | 58.8017 |
| NS1 | H-A | 46.0269 | 73.9359 | 59.9814 |
| NS1 | H-A | 52.3645 | 78.811 | 65.58775 |
| NS1 | H-A | 57.8004 | 85.2547 | 71.52755 |
| NS1 | H-A | 53.0448 | 87.2545 | 70.14965 |
| NS1 | H-A | 53.0448 | 76.9244 | 64.9846 |
| NS1 | H-A | 53.442 | 87.2545 | 70.34825 |
| NS1 | H-A | 61.2099 | 87.2545 | 74.2322 |
| NS1 | H-A | 61.5642 | 87.2545 | 74.40935 |
| NS1 | A-H | 0.0000 | 2.2079 | 1.10395 |
| NS1 | A-S | 0.0000 | 2.5622 | 1.2811 |

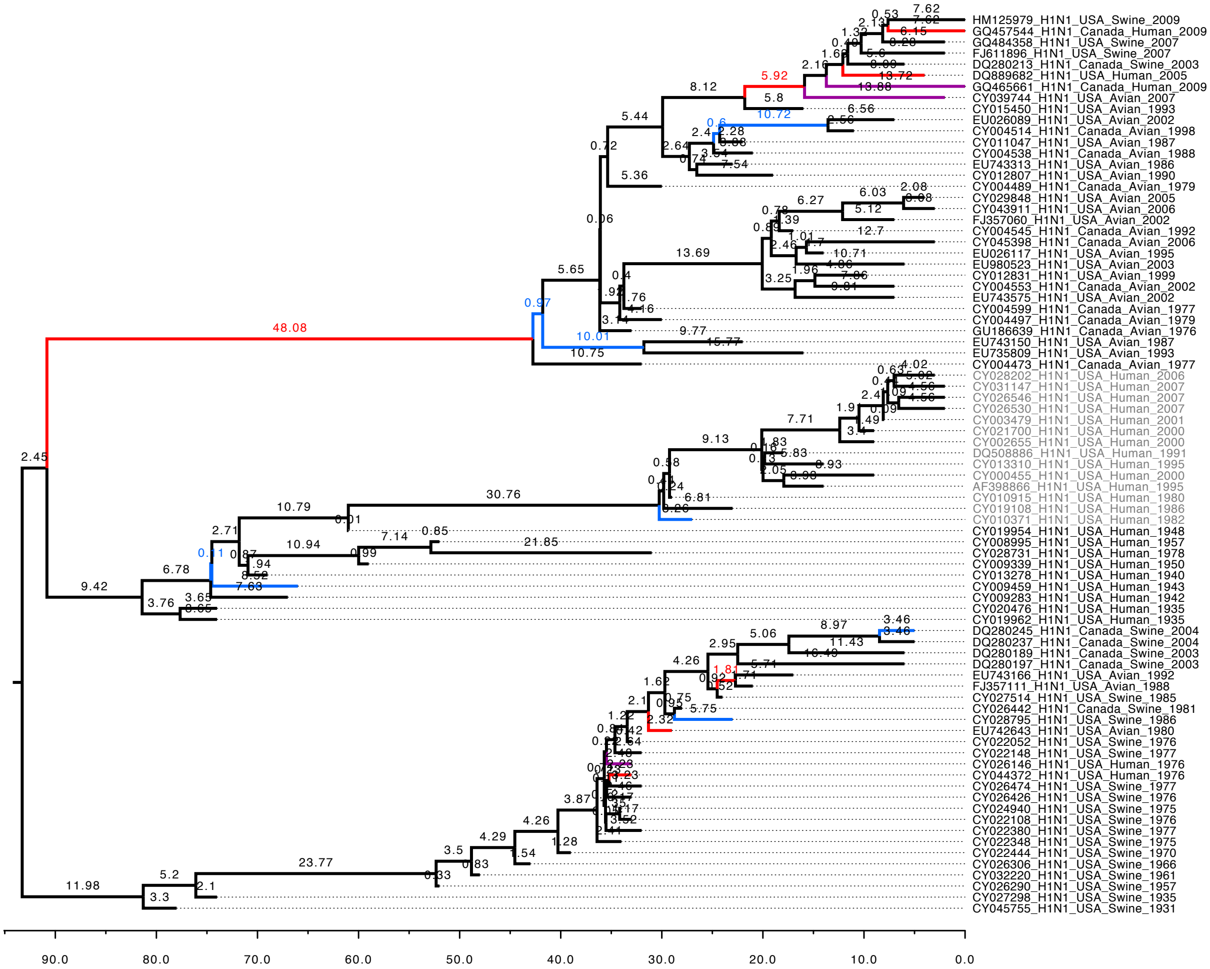


Figure S1: **Timed tree for subtype H1N1 gene PB2.** Branches are color-coded: red for a host-switch event; blue for a change in GC3 cluster; purple for a change in both host and GC3 cluster. Numbers indicate branch lengths in years. Sequences that reemerged in 1977 after a 20-year absence are indicated in gray.

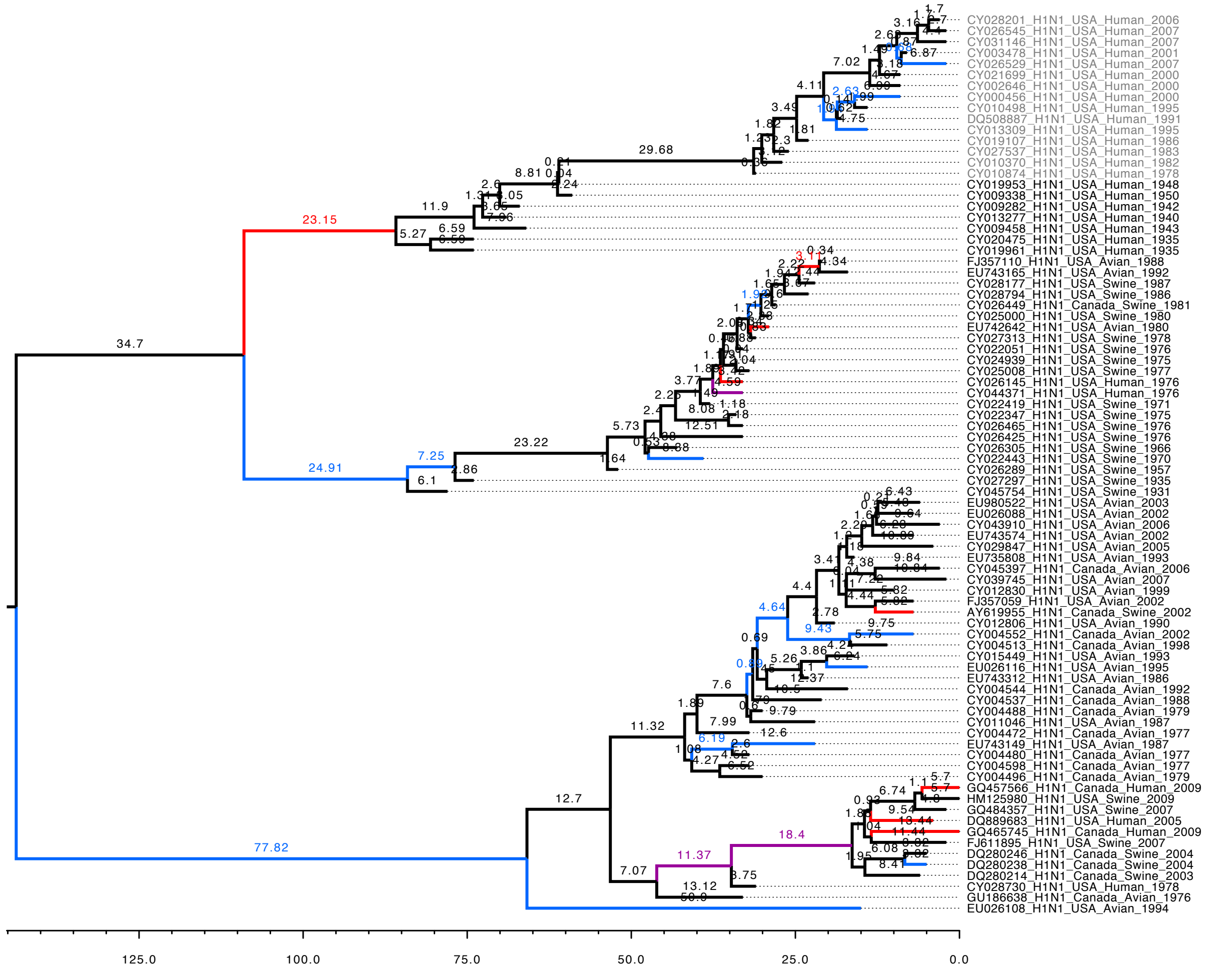


Figure S2: **Timed tree for subtype H1N1 gene PB1.** Branches are color-coded: red for a host-switch event; blue for a change in GC3 cluster; purple for a change in both host and GC3 cluster. Numbers indicate branch lengths in years. Sequences that reemerged in 1977 after a 20-year absence are indicated in gray.

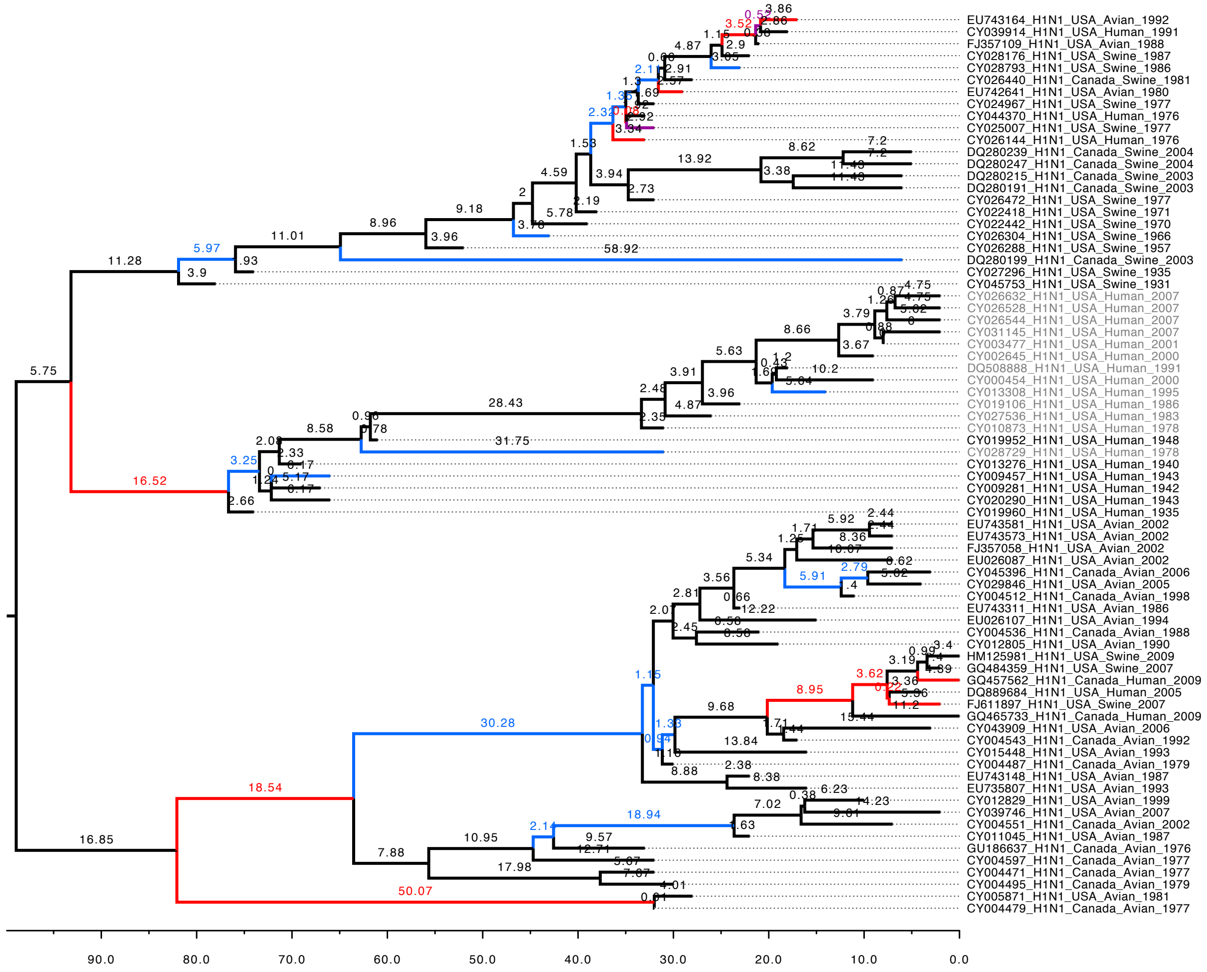


Figure S3: **Timed tree for subtype H1N1 gene PA.** Branches are color-coded: red for a host-switch event; blue for a change in GC3 cluster; purple for a change in both host and GC3 cluster. Numbers indicate branch lengths in years. Sequences that reemerged in 1977 after a 20-year absence are indicated in gray.

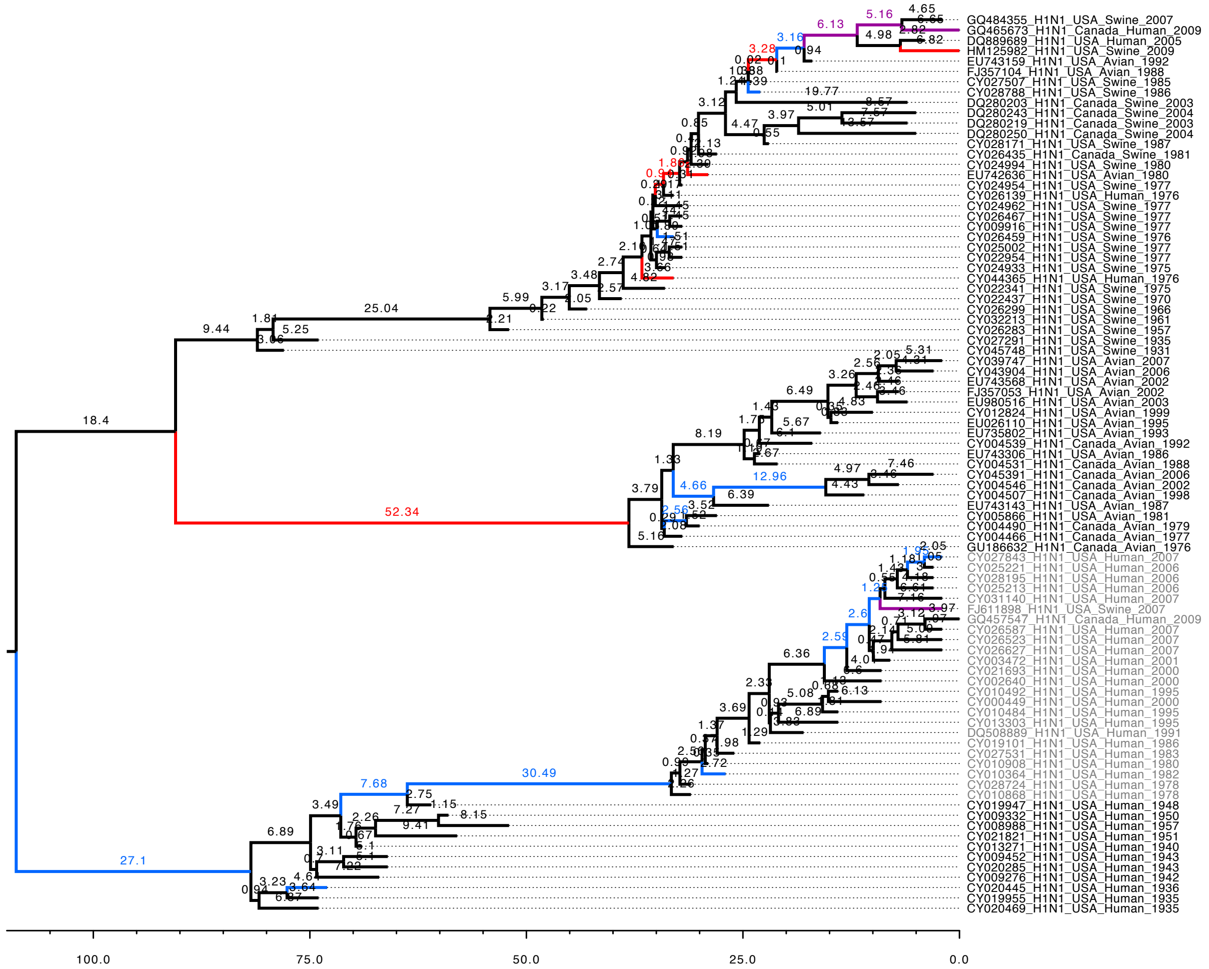


Figure S4: **Timed tree for subtype H1N1 gene HA.** Branches are color-coded: red for a host-switch event; blue for a change in GC3 cluster; purple for a change in both host and GC3 cluster. Numbers indicate branch lengths in years. Sequences that reemerged in 1977 after a 20-year absence are indicated in gray.

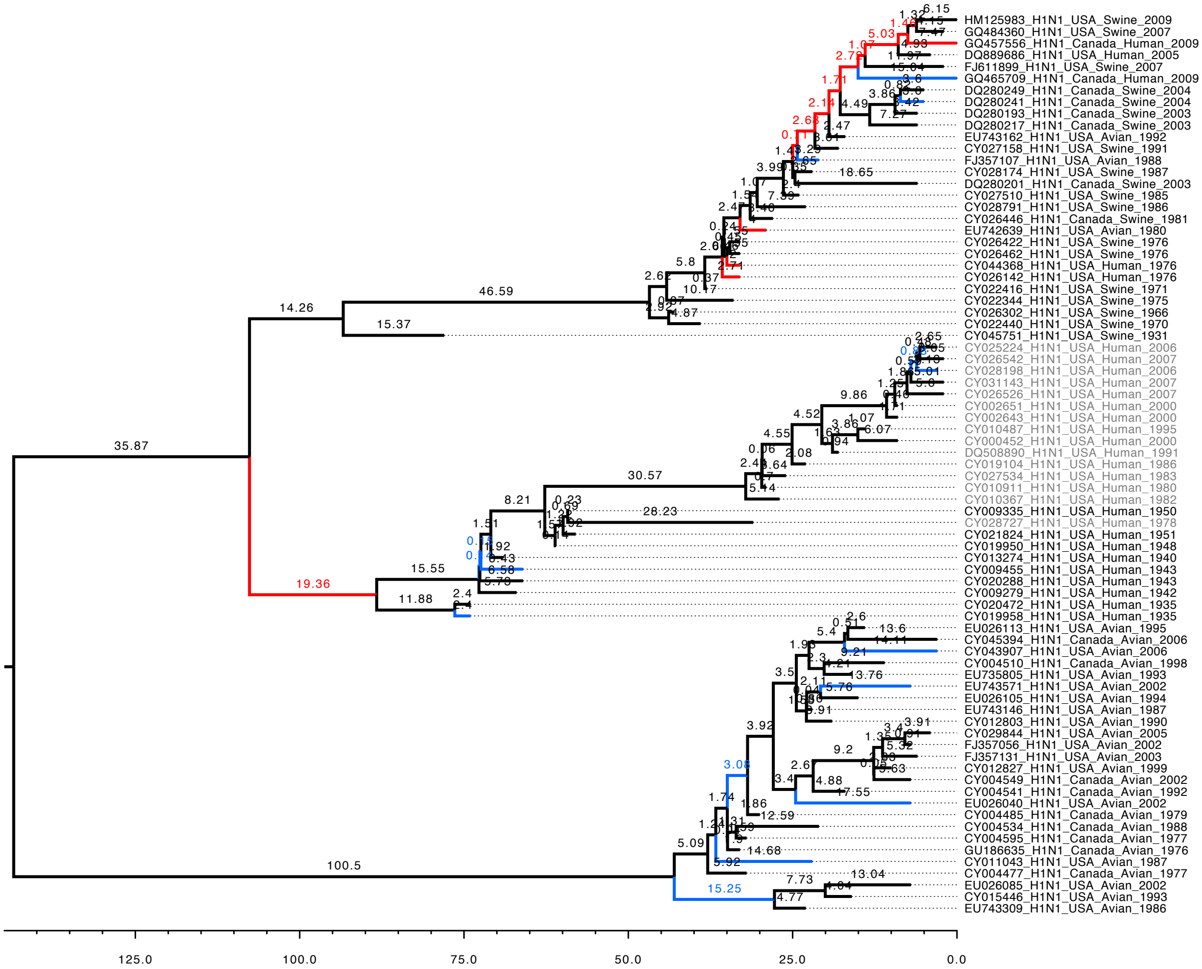


Figure S5: **Timed tree for subtype H1N1 gene NP.** Branches are color-coded: red for a host-switch event; blue for a change in GC3 cluster; purple for a change in both host and GC3 cluster. Numbers indicate branch lengths in years. Sequences that reemerged in 1977 after a 20-year absence are indicated in gray.

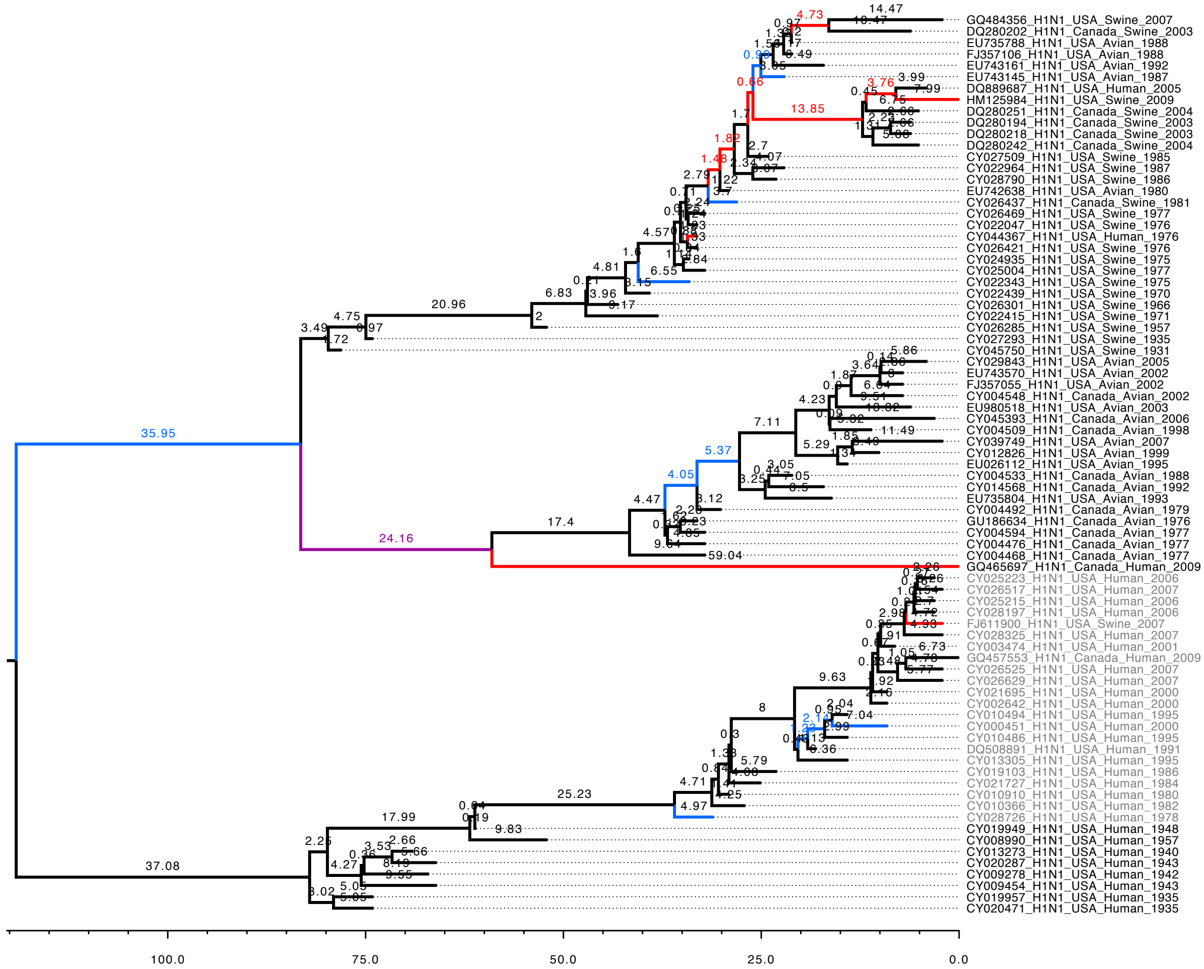


Figure S6: **Timed tree for subtype H1N1 gene NA.** Branches are color-coded: red for a host-switch event; blue for a change in GC3 cluster; purple for a change in both host and GC3 cluster. Numbers indicate branch lengths in years. Sequences that reemerged in 1977 after a 20-year absence are indicated in gray.

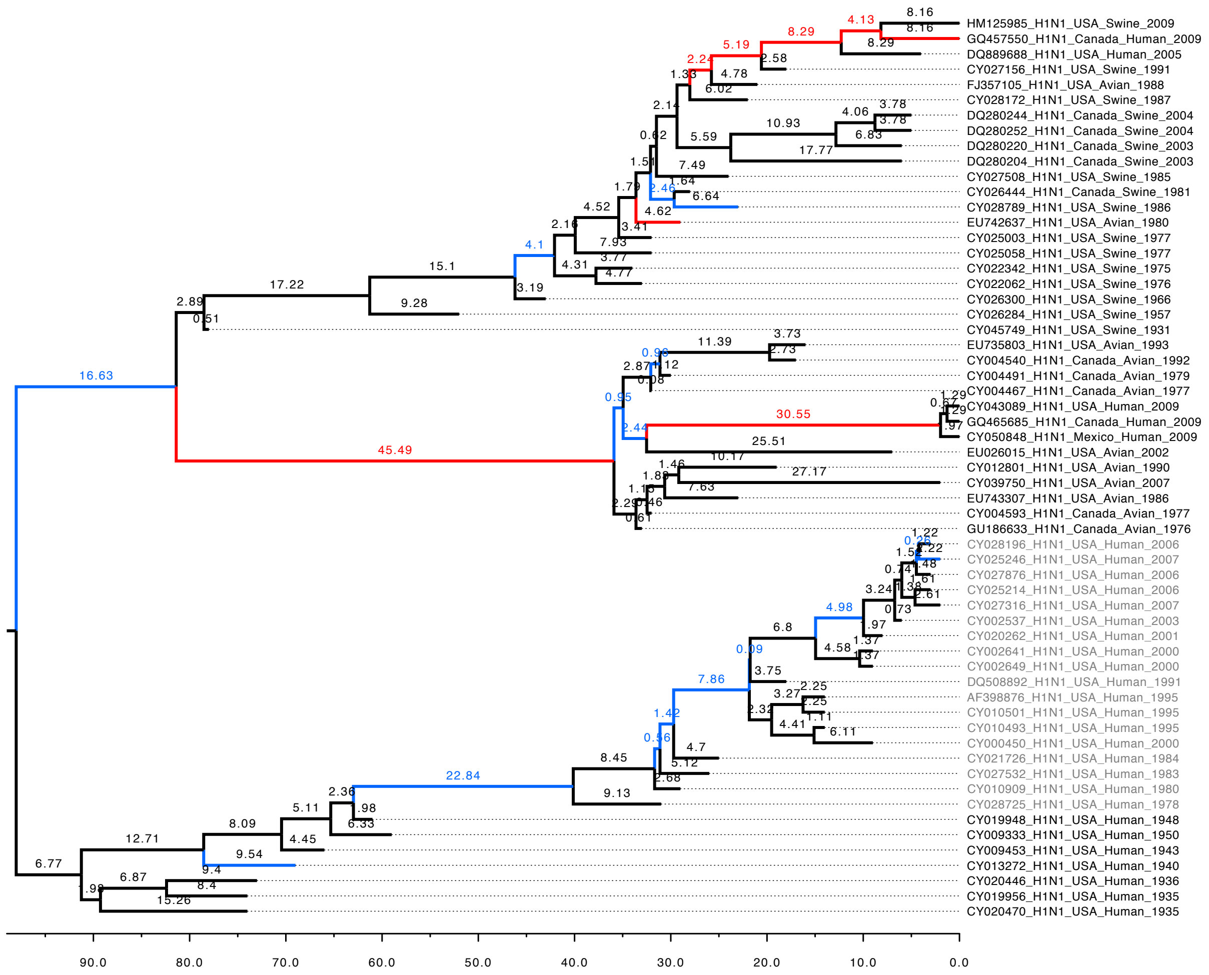


Figure S7: **Timed tree for subtype H1N1 gene M2.** Branches are color-coded: red for a host-switch event; blue for a change in GC3 cluster; purple for a change in both host and GC3 cluster. Numbers indicate branch lengths in years. Sequences that reemerged in 1977 after a 20-year absence are indicated in gray.

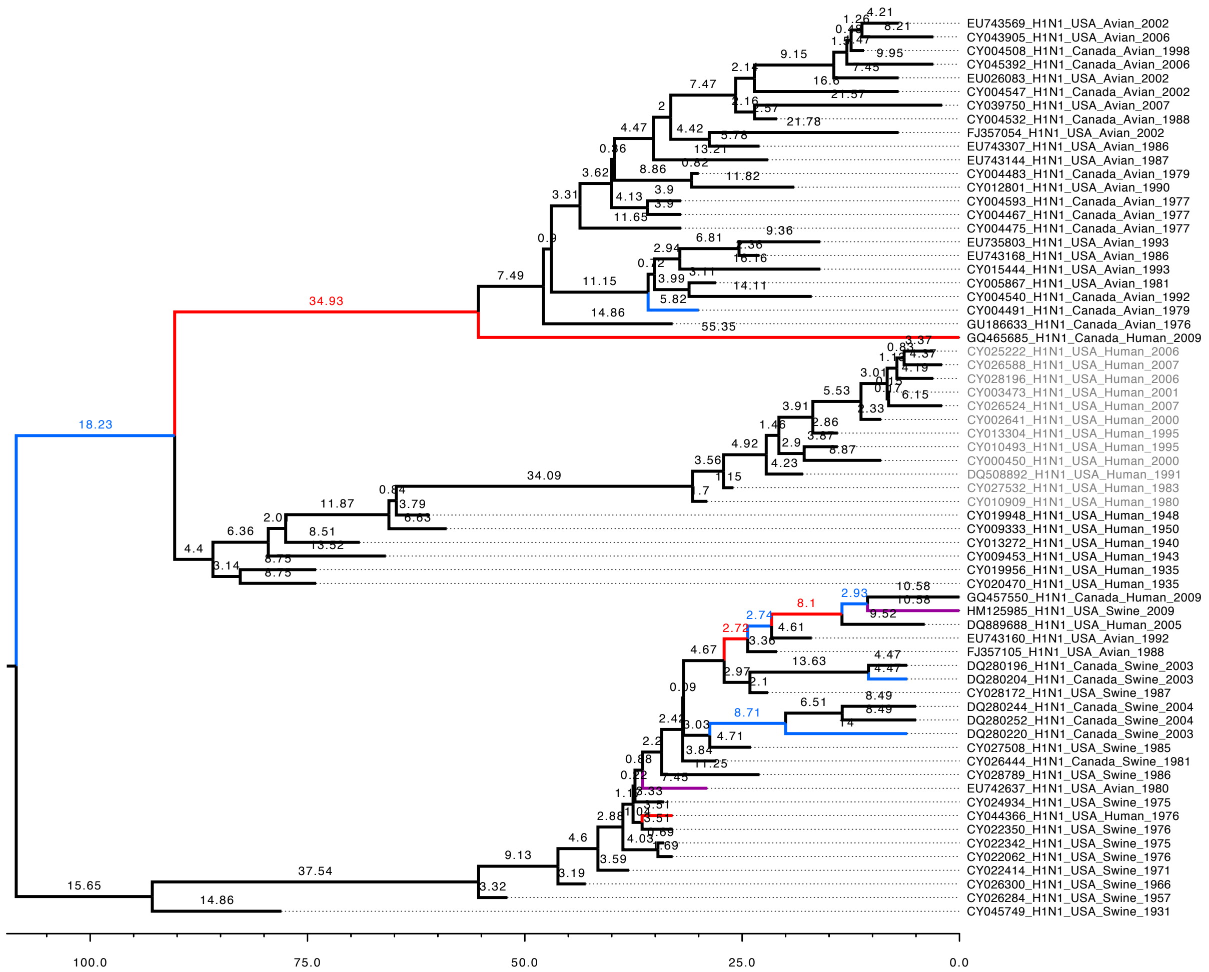


Figure S8: **Timed tree for subtype H1N1 gene M1.** Branches are color-coded: red for a host-switch event; blue for a change in GC3 cluster; purple for a change in both host and GC3 cluster. Numbers indicate branch lengths in years. Sequences that reemerged in 1977 after a 20-year absence are indicated in gray.

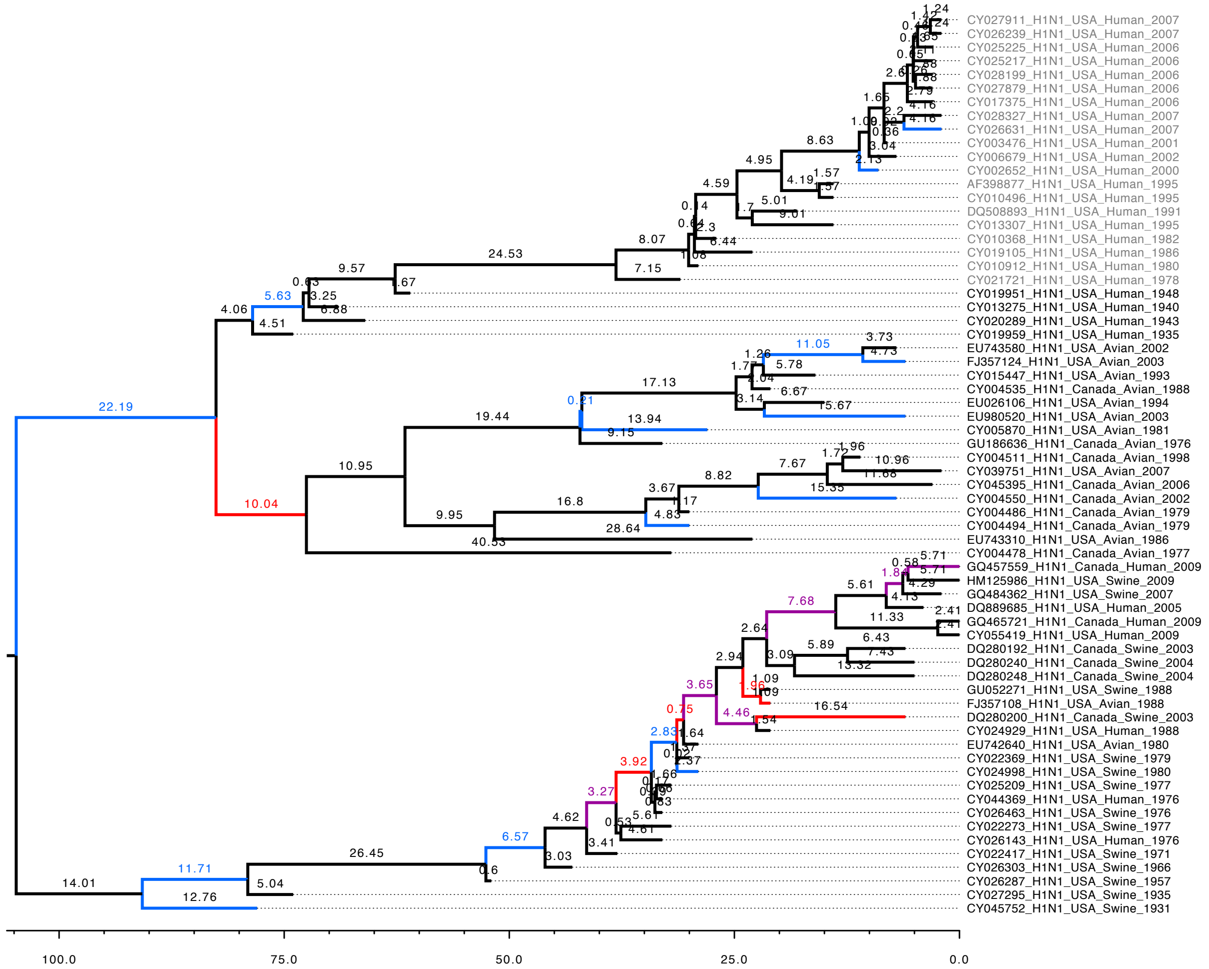


Figure S9: **Timed tree for subtype H1N1 gene NS2.** Branches are color-coded: red for a host-switch event; blue for a change in GC3 cluster; purple for a change in both host and GC3 cluster. Numbers indicate branch lengths in years. Sequences that reemerged in 1977 after a 20-year absence are indicated in gray.

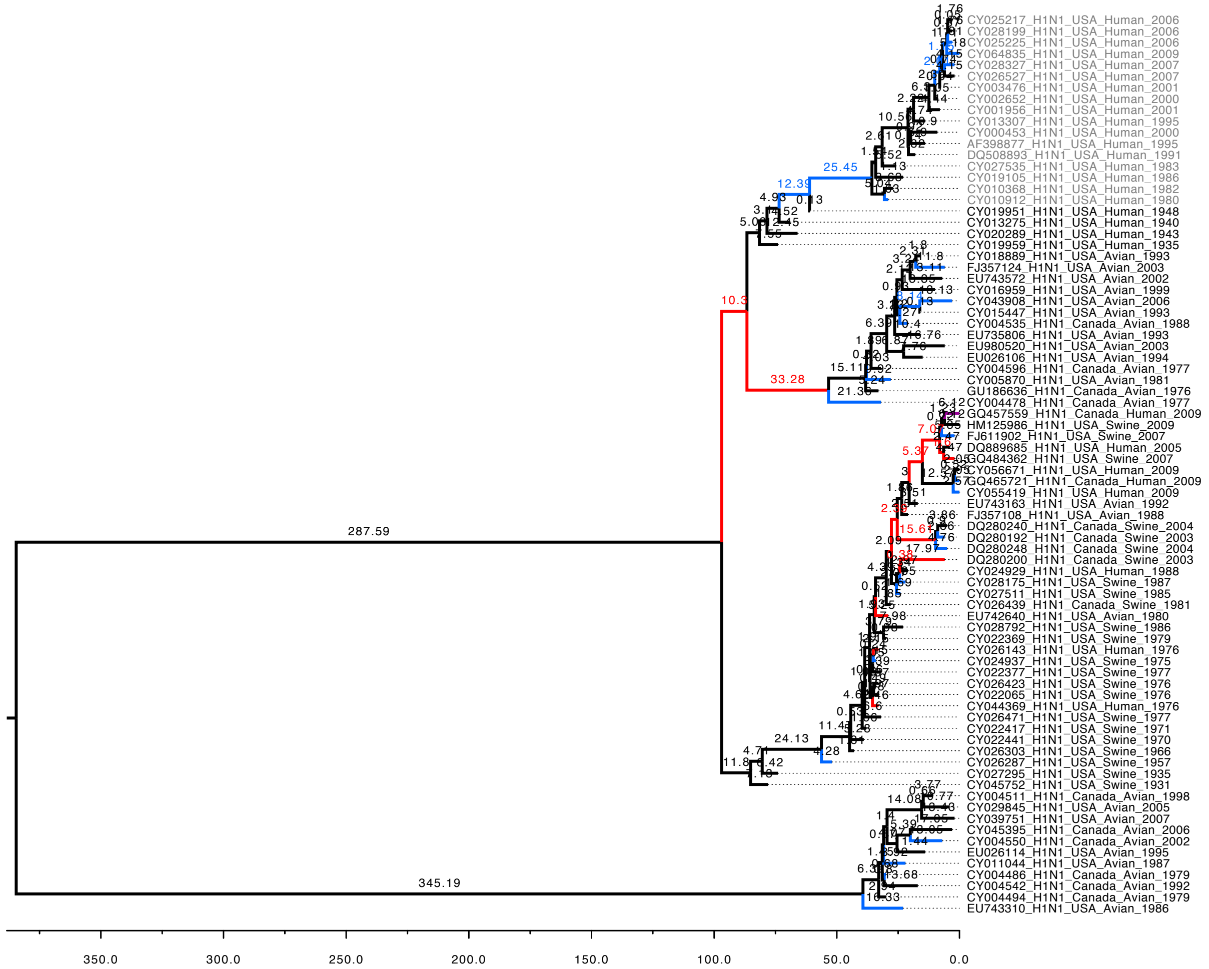


Figure S10: **Timed tree for subtype H1N1 gene NS1.** Branches are color-coded: red for a host-switch event; blue for a change in GC3 cluster; purple for a change in both host and GC3 cluster. Numbers indicate branch lengths in years. Sequences that reemerged in 1977 after a 20-year absence are indicated in gray.

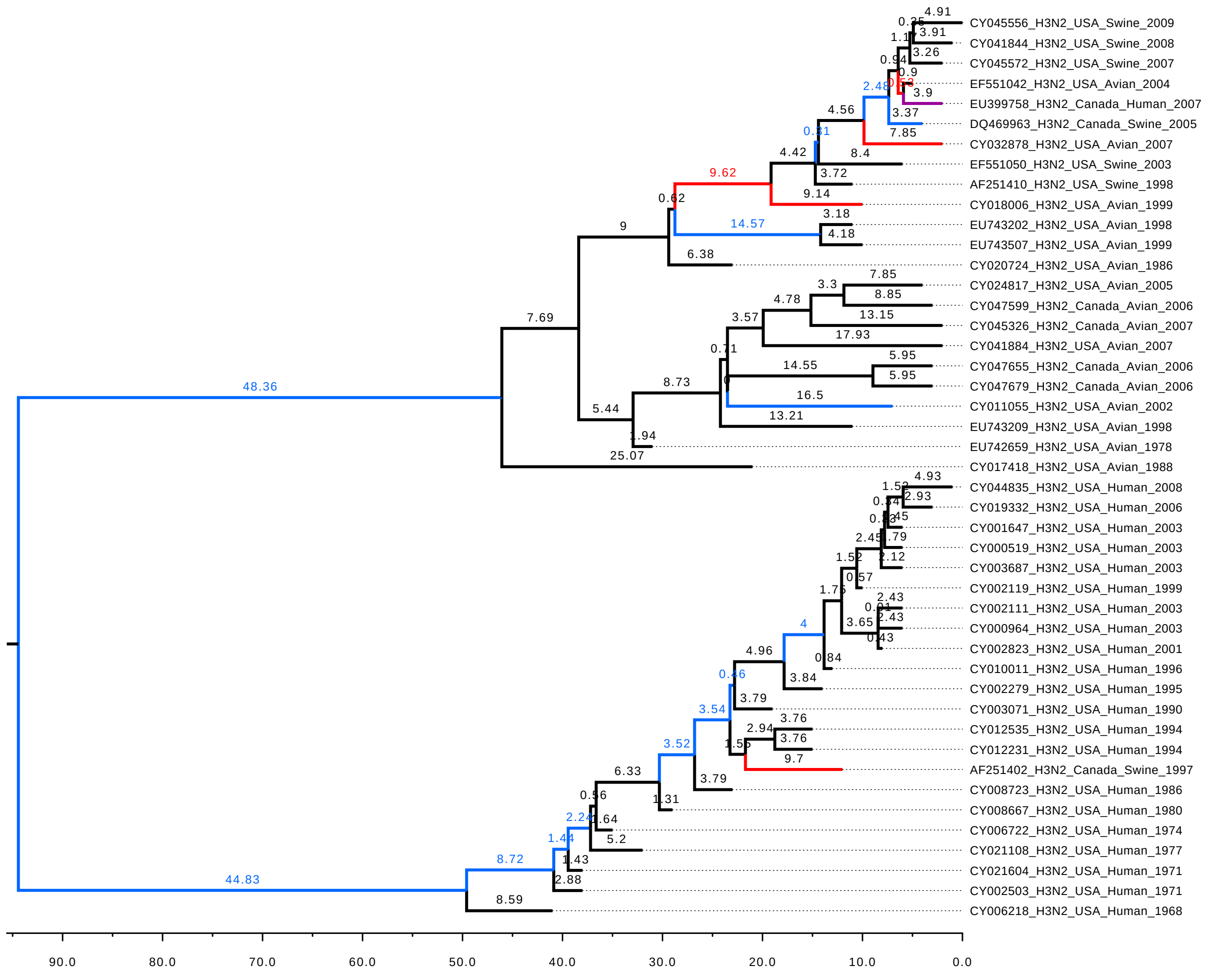


Figure S11: **Timed tree for subtype H3N2 gene PB2.** Branches are color-coded: red for a host-switch event; blue for a change in GC3 cluster; purple for a change in both host and GC3 cluster. Numbers indicate branch lengths in years.

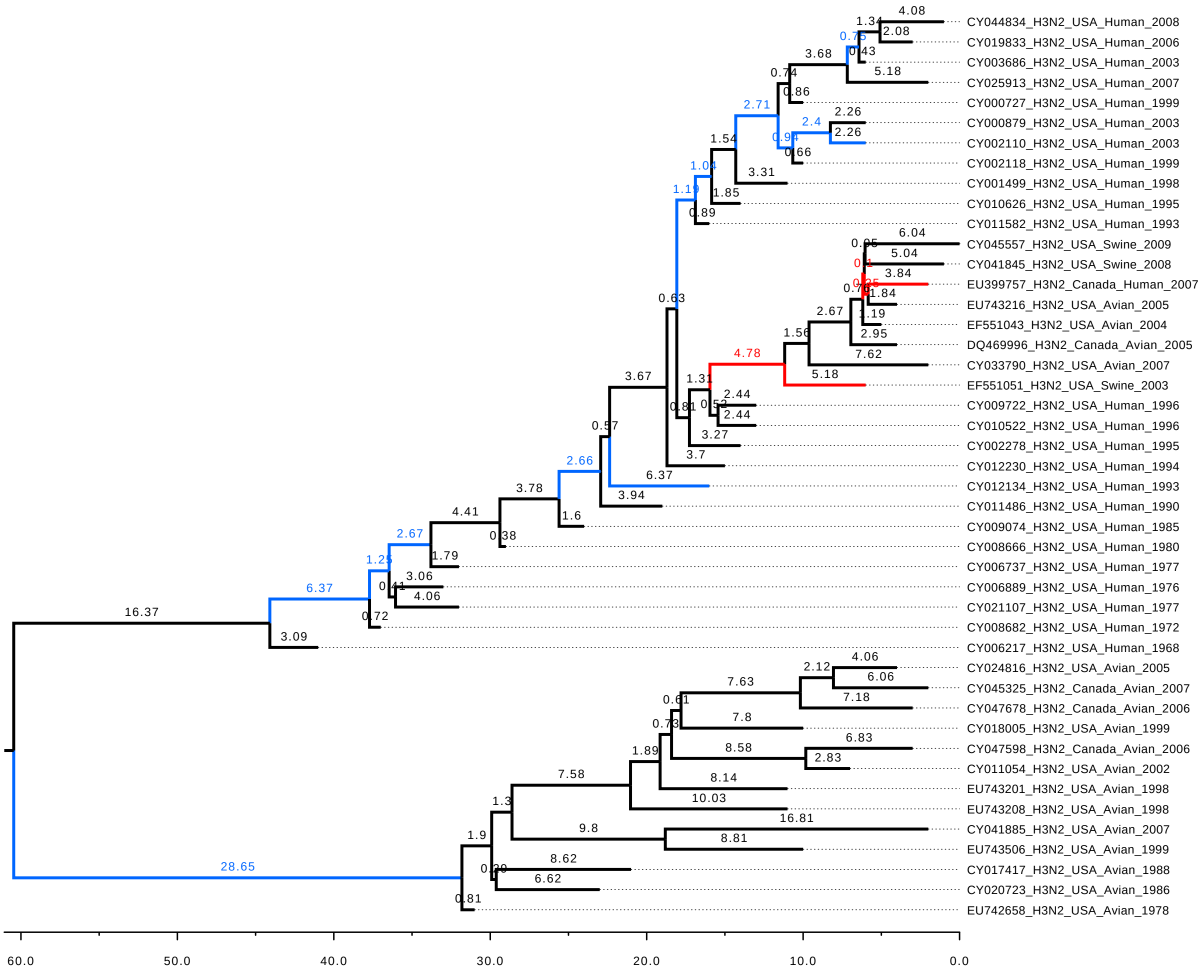


Figure S12: **Timed tree for subtype H3N2 gene PB1.** Branches are color-coded: red for a host-switch event; blue for a change in GC3 cluster; purple for a change in both host and GC3 cluster. Numbers indicate branch lengths in years.

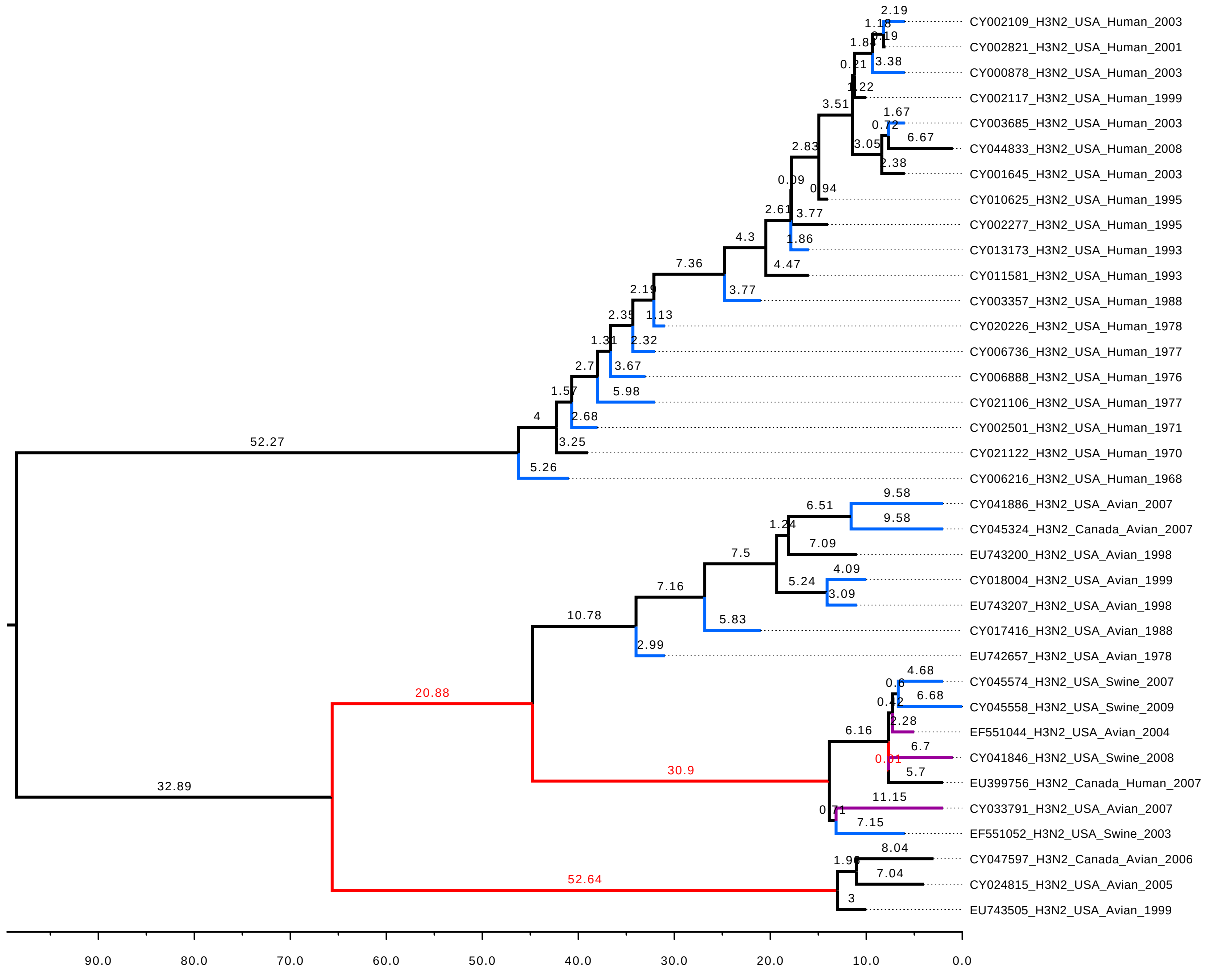


Figure S13: **Timed tree for subtype H3N2 gene PA.** Branches are color-coded: red for a host-switch event; blue for a change in GC3 cluster; purple for a change in both host and GC3 cluster. Numbers indicate branch lengths in years.

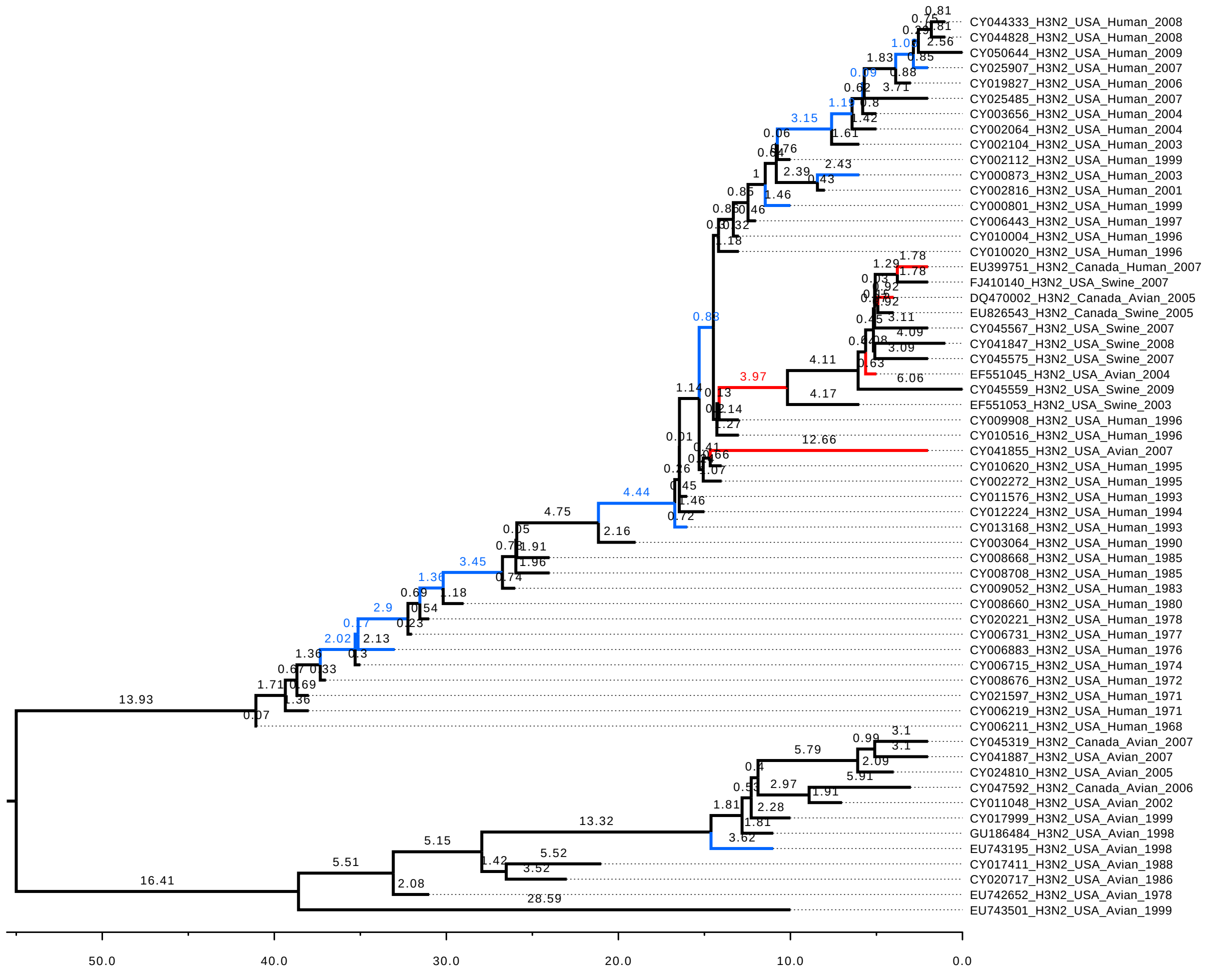


Figure S14: **Timed tree for subtype H3N2 gene HA.** Branches are color-coded: red for a host-switch event; blue for a change in GC3 cluster; purple for a change in both host and GC3 cluster. Numbers indicate branch lengths in years.

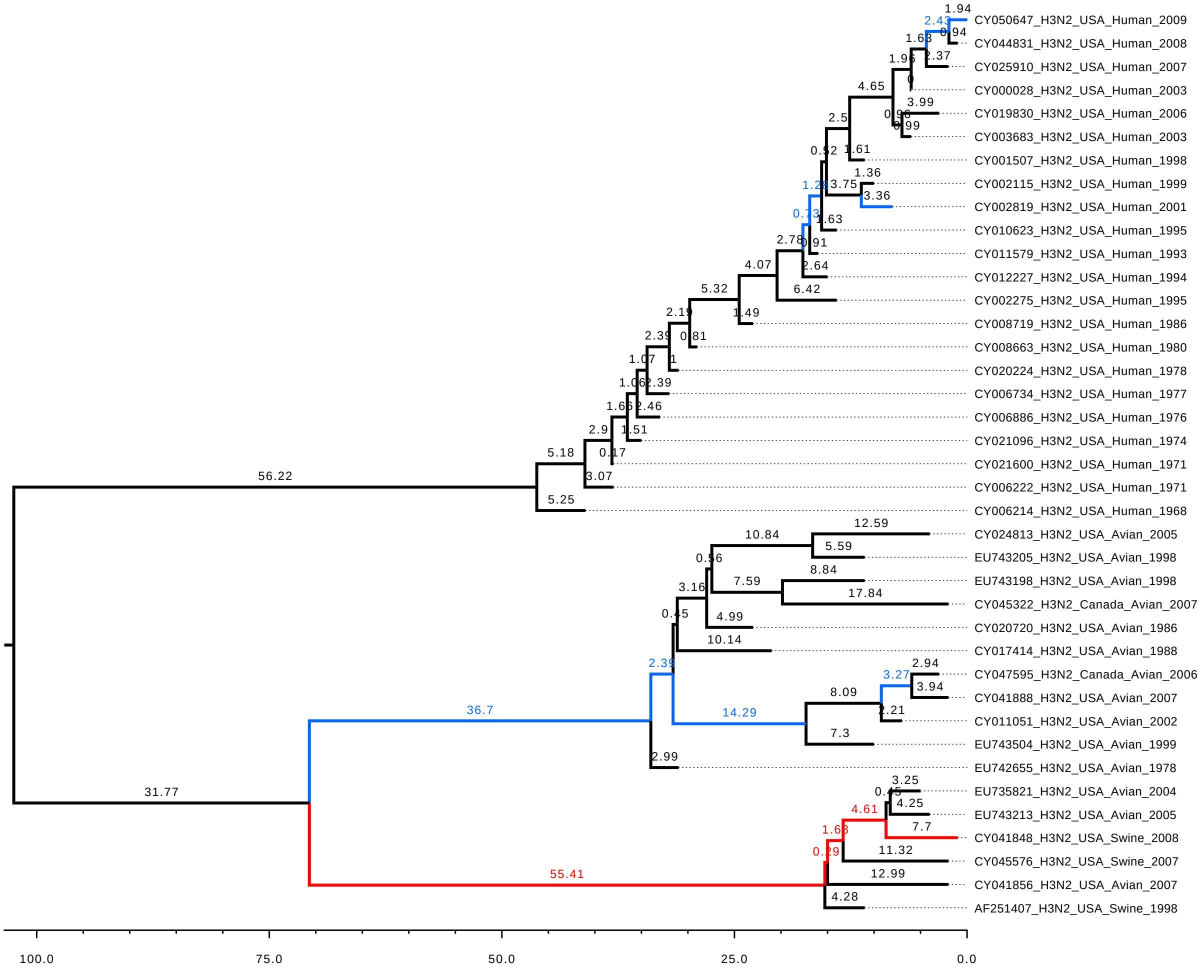


Figure S15: **Timed tree for subtype H3N2 gene NP.** Branches are color-coded: red for a host-switch event; blue for a change in GC3 cluster; purple for a change in both host and GC3 cluster. Numbers indicate branch lengths in years.

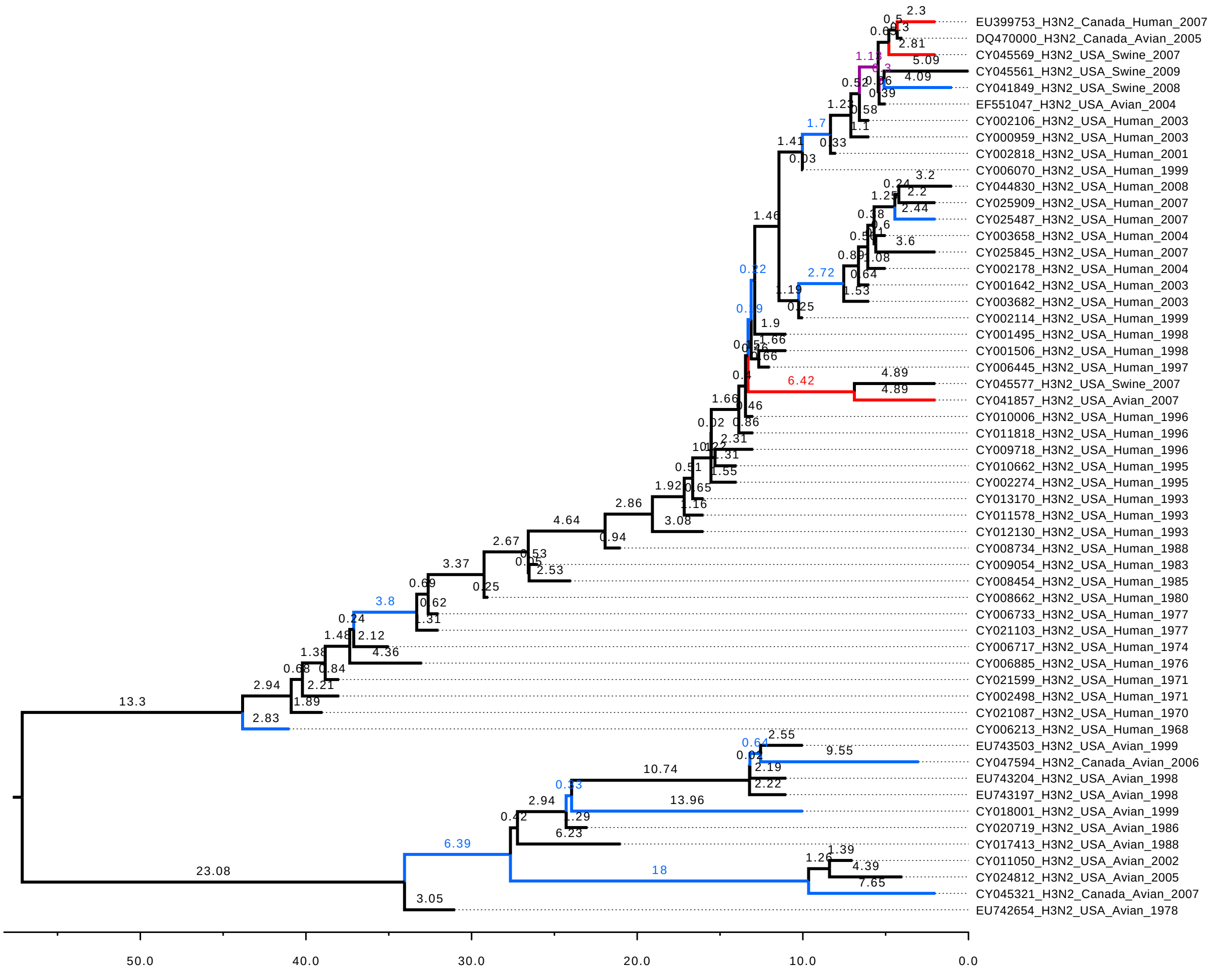


Figure S16: **Timed tree for subtype H3N2 gene NA.** Branches are color-coded: red for a host-switch event; blue for a change in GC3 cluster; purple for a change in both host and GC3 cluster. Numbers indicate branch lengths in years.

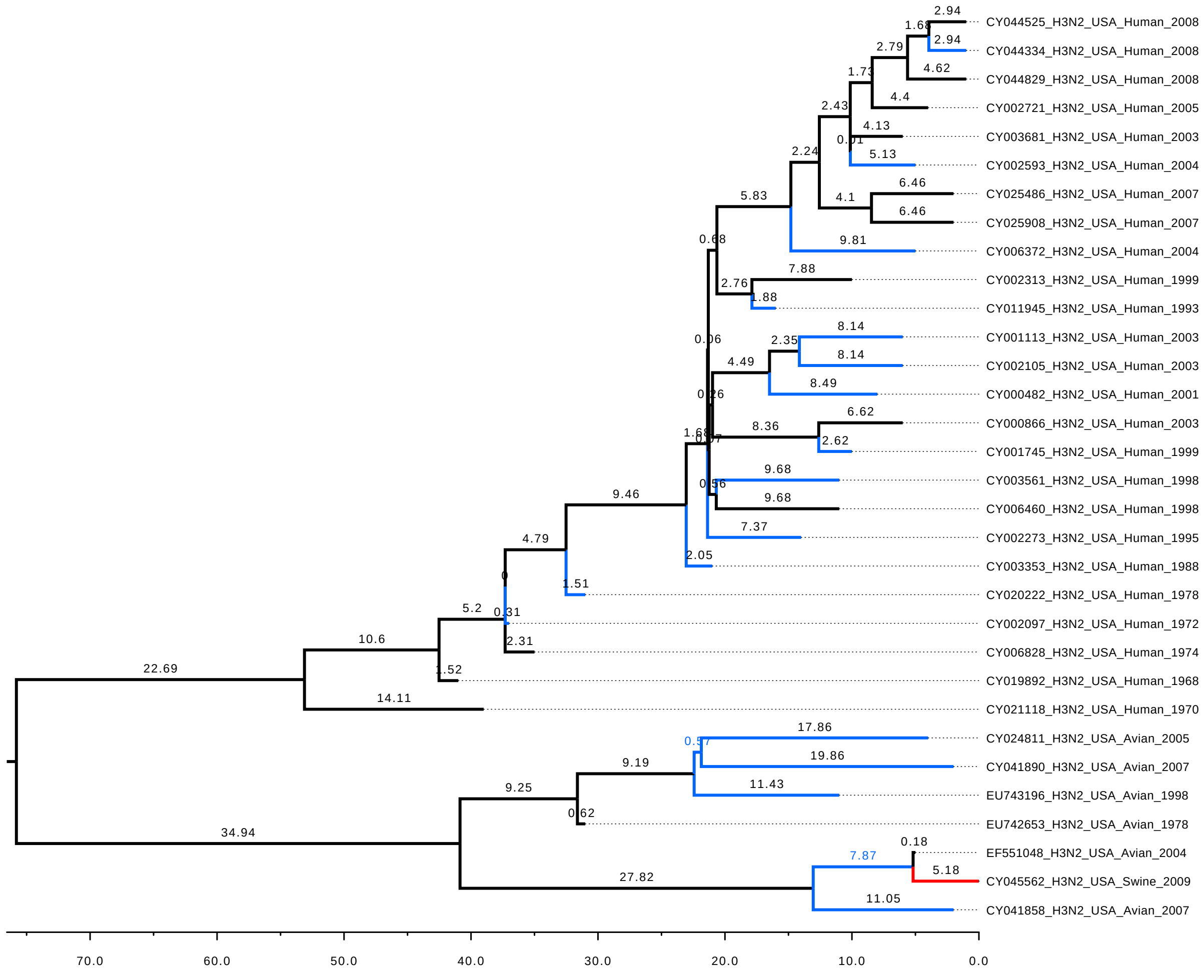


Figure S17: **Timed tree for subtype H3N2 gene M2.** Branches are color-coded: red for a host-switch event; blue for a change in GC3 cluster; purple for a change in both host and GC3 cluster. Numbers indicate branch lengths in years.

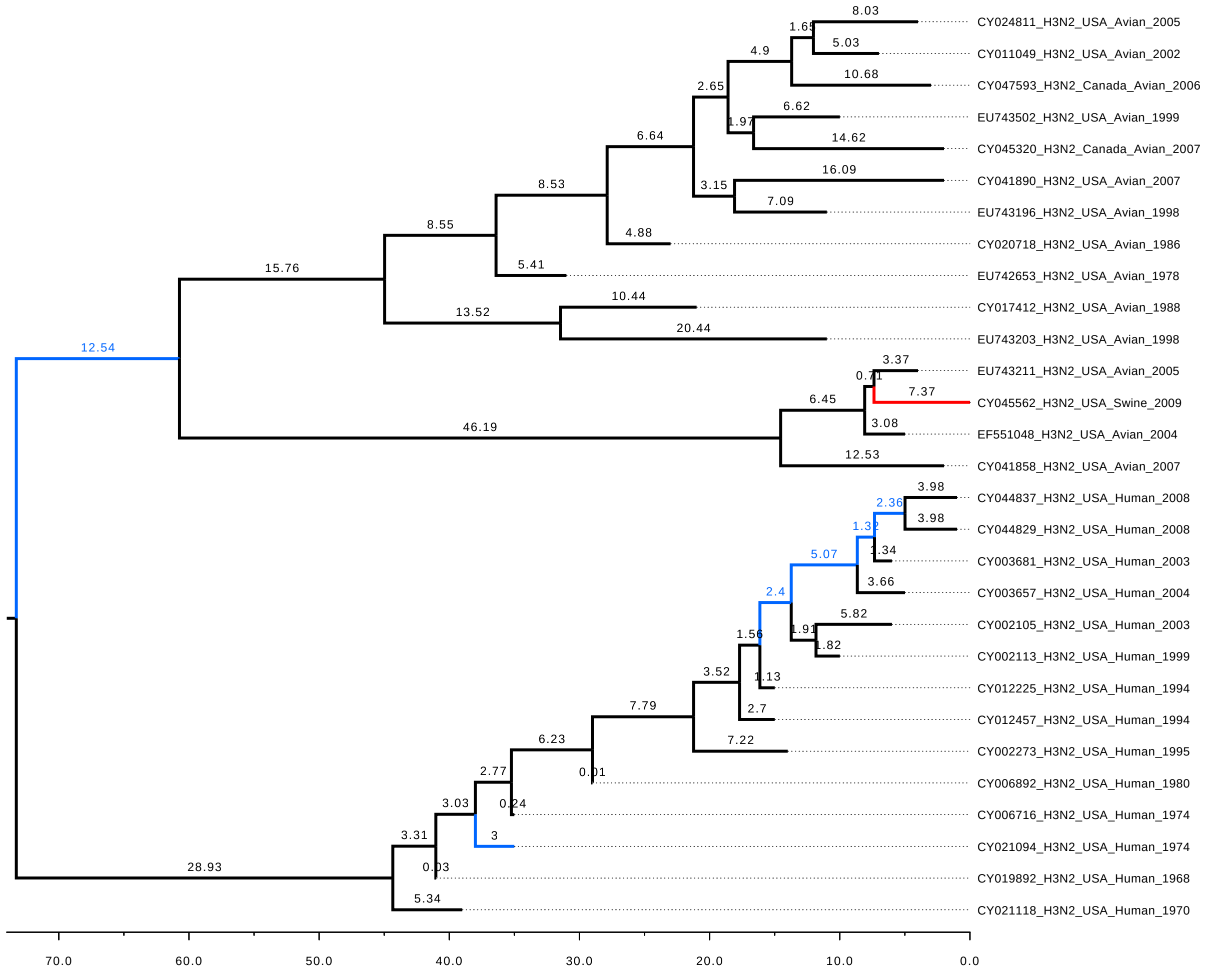


Figure S18: **Timed tree for subtype H3N2 gene M1.** Branches are color-coded: red for a host-switch event; blue for a change in GC3 cluster; purple for a change in both host and GC3 cluster. Numbers indicate branch lengths in years.

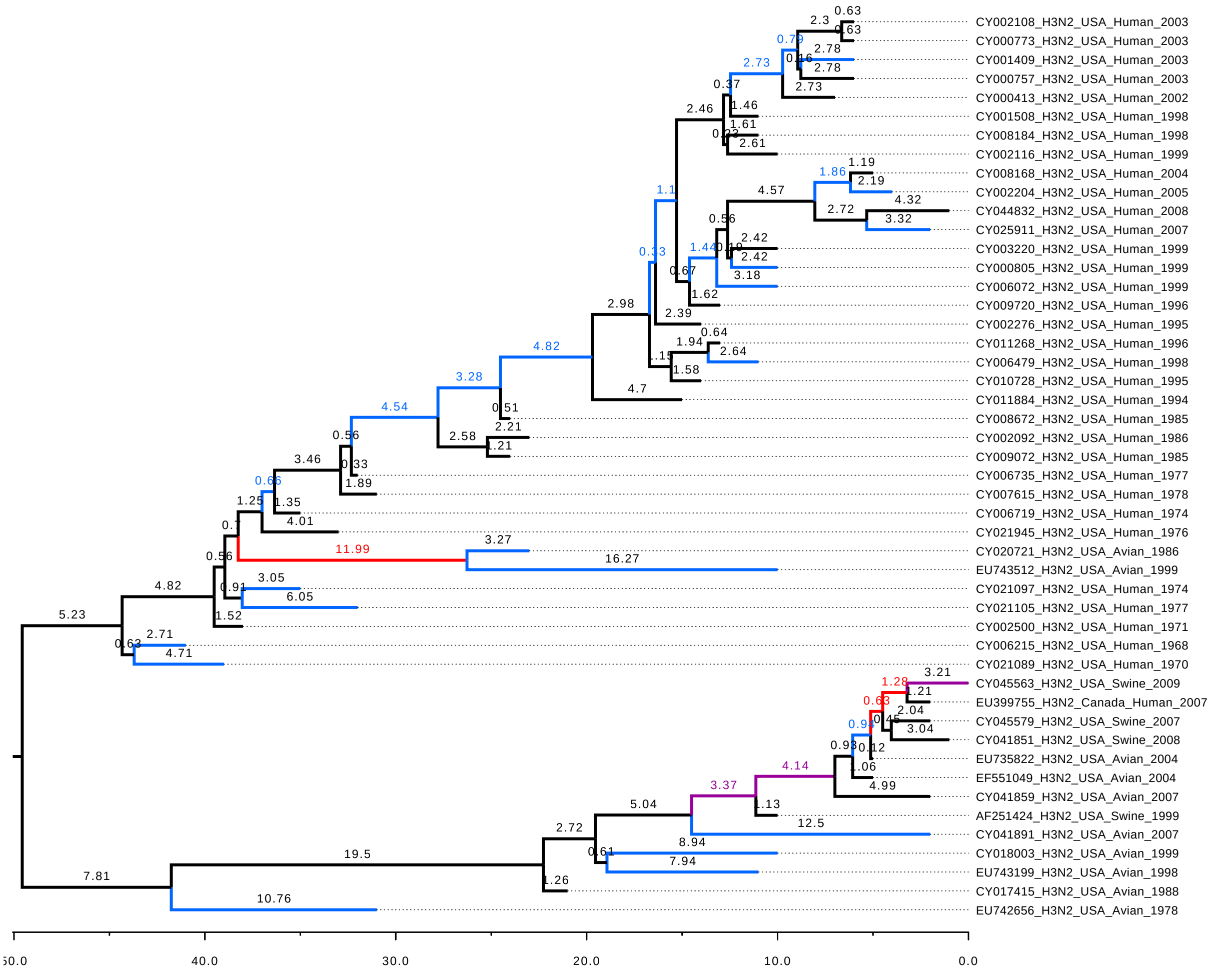


Figure S19: **Timed tree for subtype H3N2 gene NS2.** Branches are color-coded: red for a host-switch event; blue for a change in GC3 cluster; purple for a change in both host and GC3 cluster. Numbers indicate branch lengths in years.

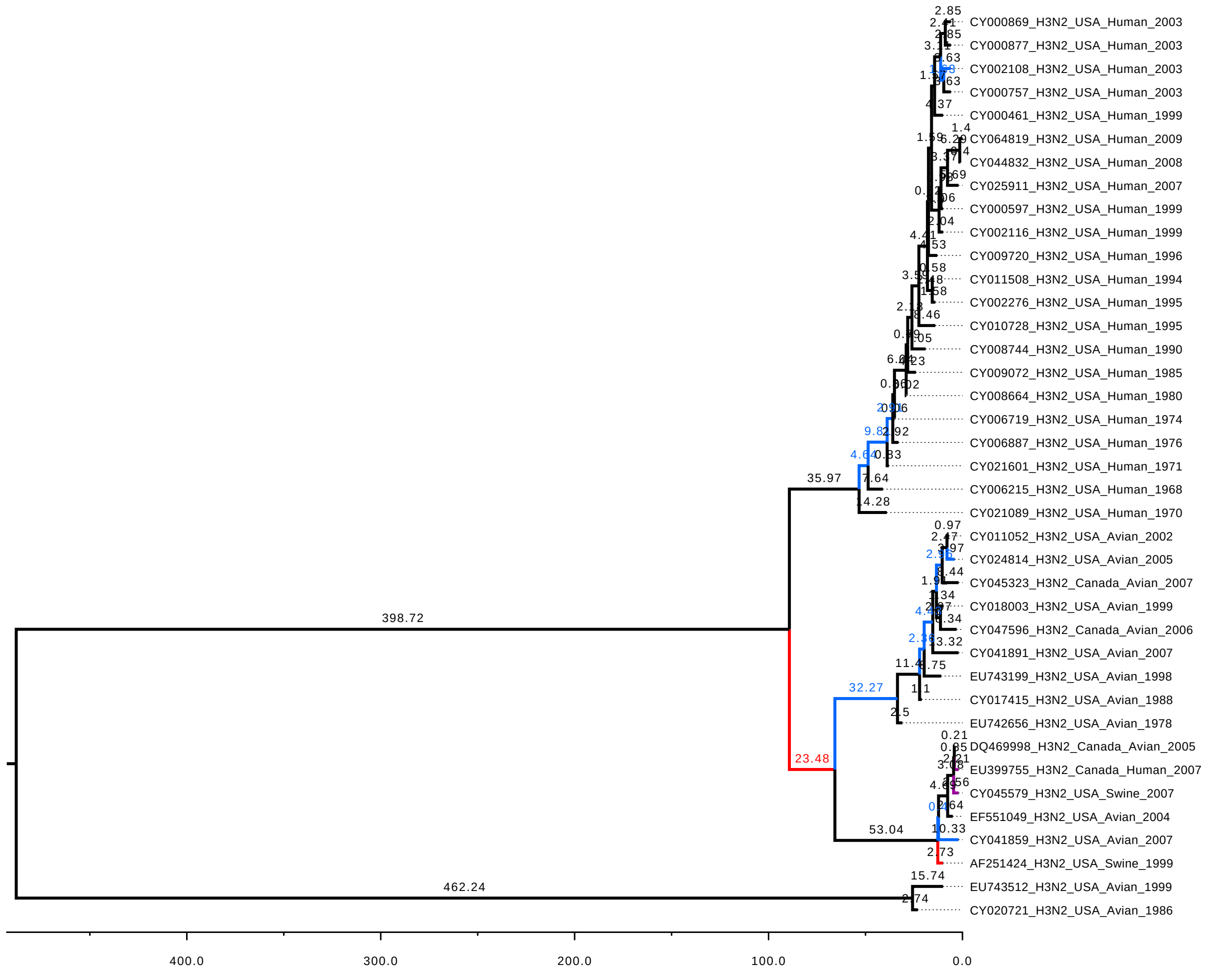


Figure S20: **Timed tree for subtype H3N2 gene NS1.** Branches are color-coded: red for a host-switch event; blue for a change in GC3 cluster; purple for a change in both host and GC3 cluster. Numbers indicate branch lengths in years.

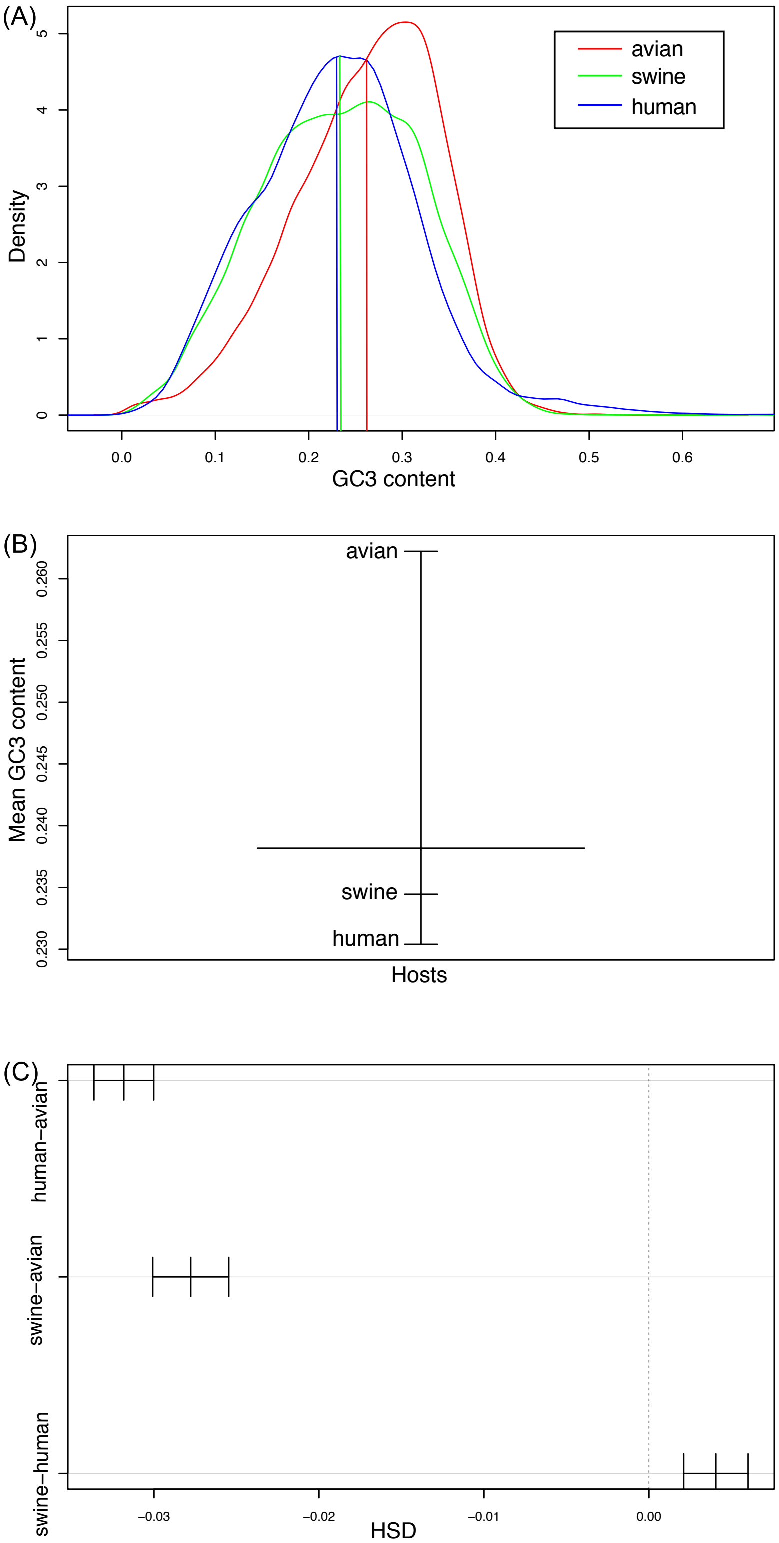


Figure S21: **GC3 composition of the host species.** (A) Density plots of GC3 contents of avian (red), swine (green) and human (blue) hosts; vertical lines represent mean values for each density. (B) Factor effects. (C) Graphical representation of Tukey's Honestly Significant Differences (HSD).

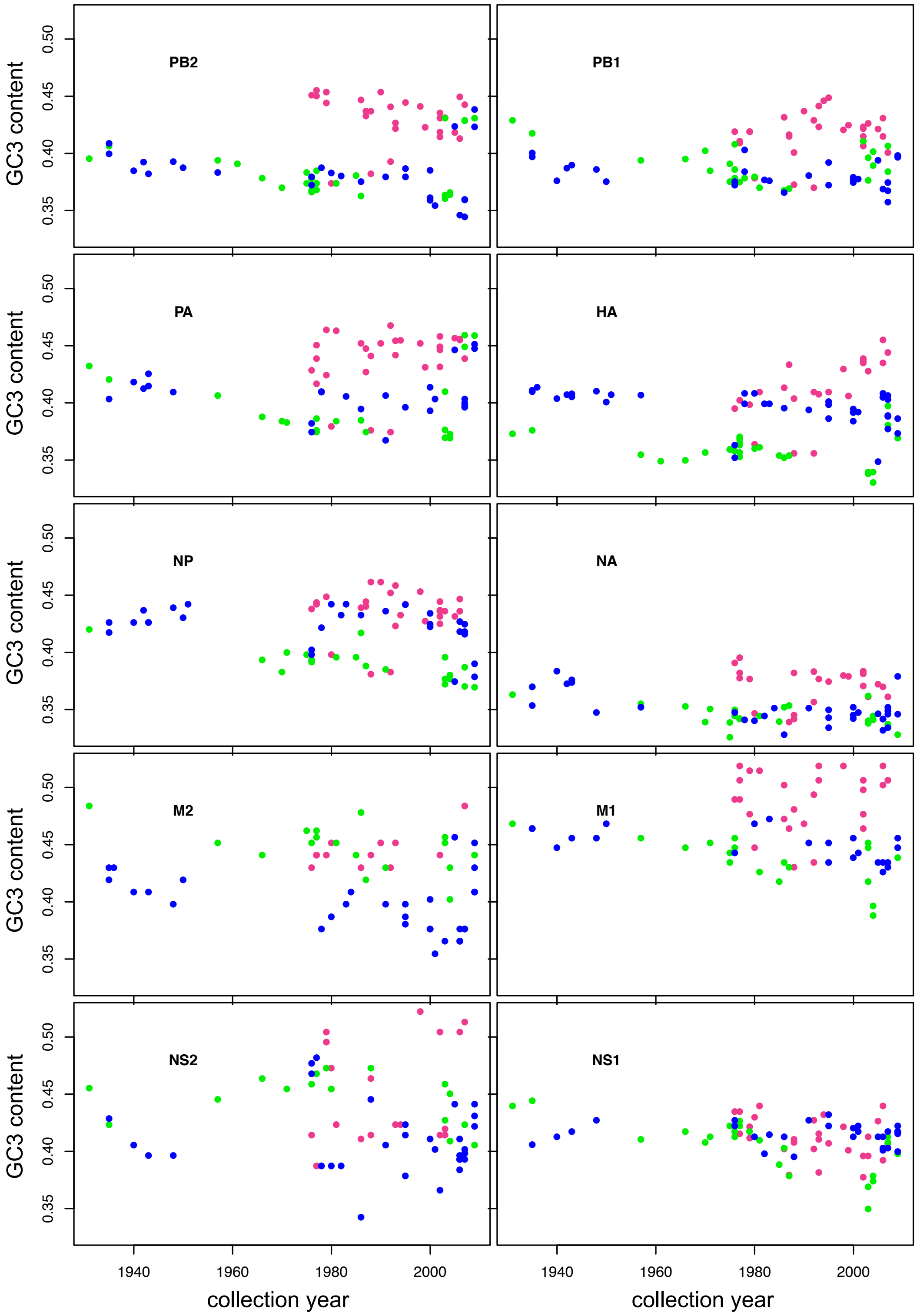


Figure S22: **GC3 composition of H1N1 viruses as a function of collection dates.** Hosts are color-coded: avian in purple, human in blue and swine in green.

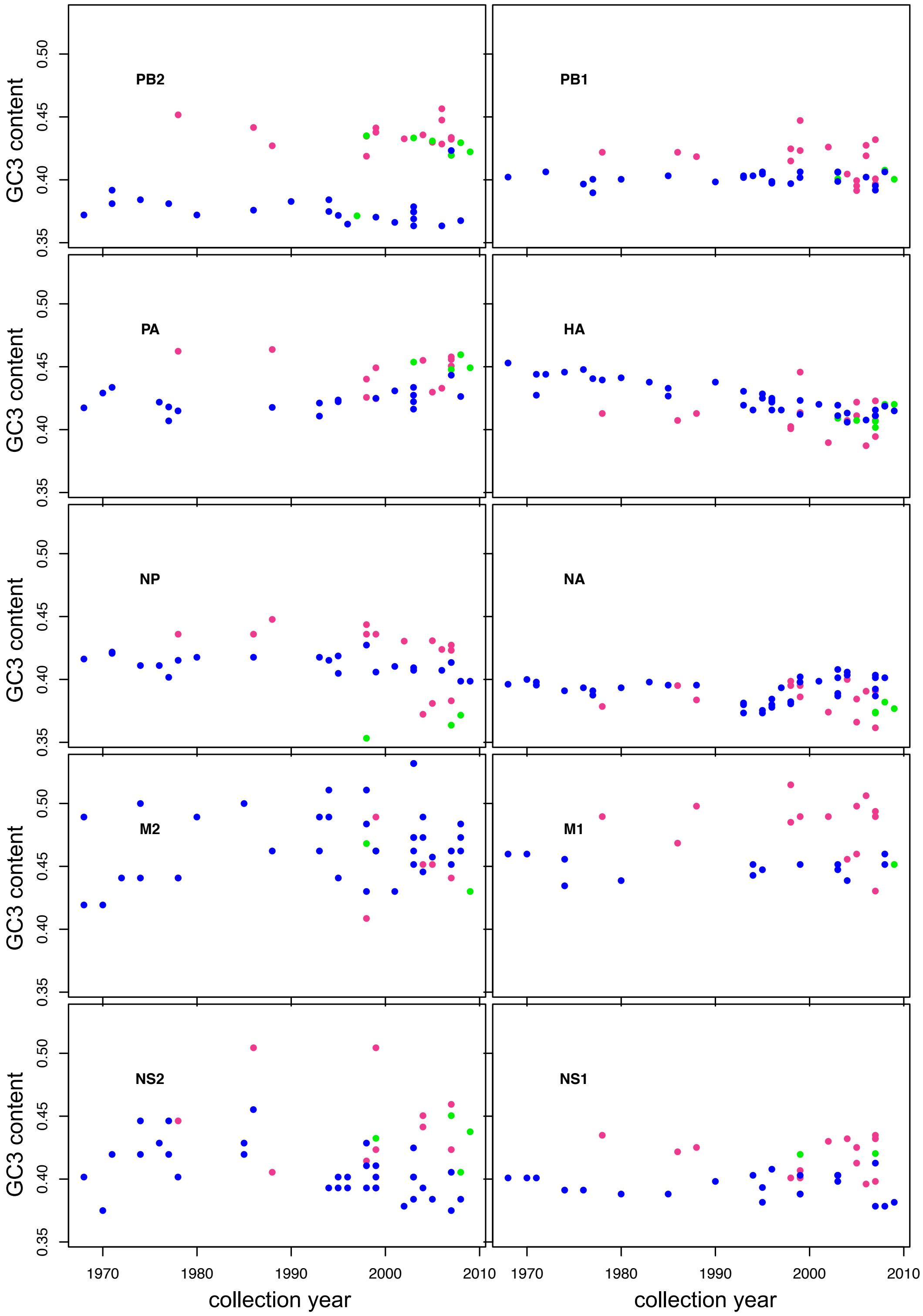


Figure S23: **GC3 composition of H3N2 viruses as a function of collection dates.** Hosts are color-coded: avian in purple, human in blue and swine in green.

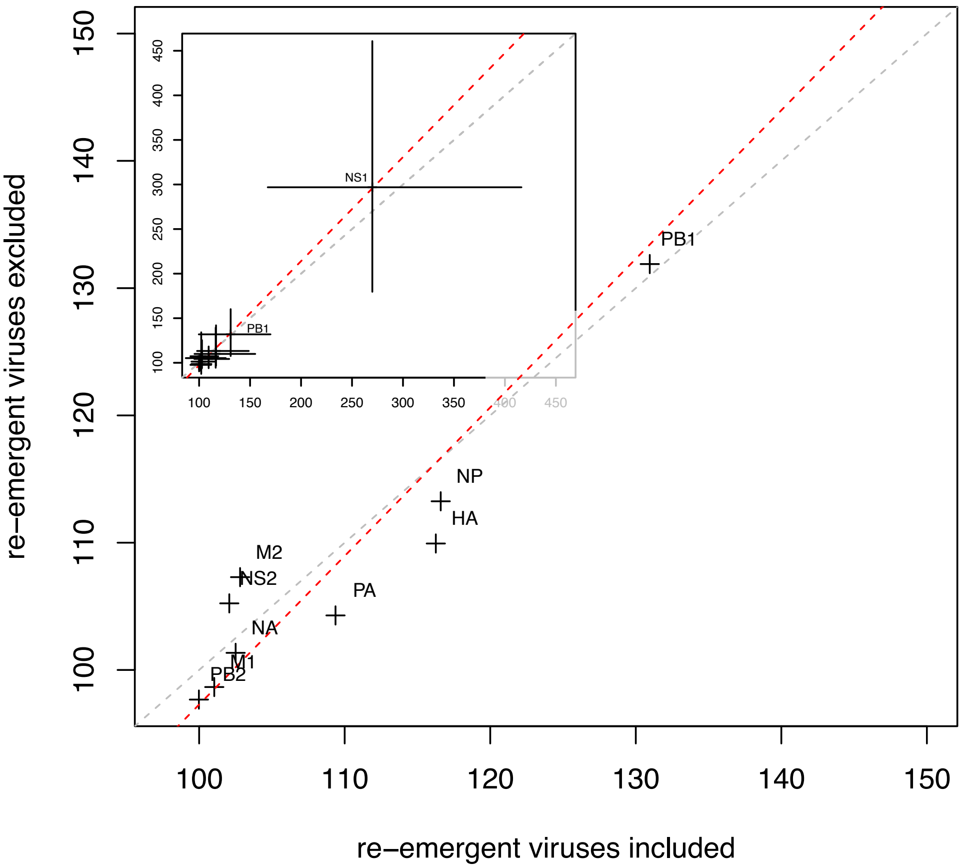


Figure S24: **Comparison of root age for H1N1 viruses with and without re-emergent strains.** The gray line represents the first bisector (line of equation $y = x$), while the red line represents the linear fit to the data. Scale on both axes is in years before 2009. Insert shows the results for all ten 'canonical' genes, with bars representing the 95% Highest Posterior Densities.

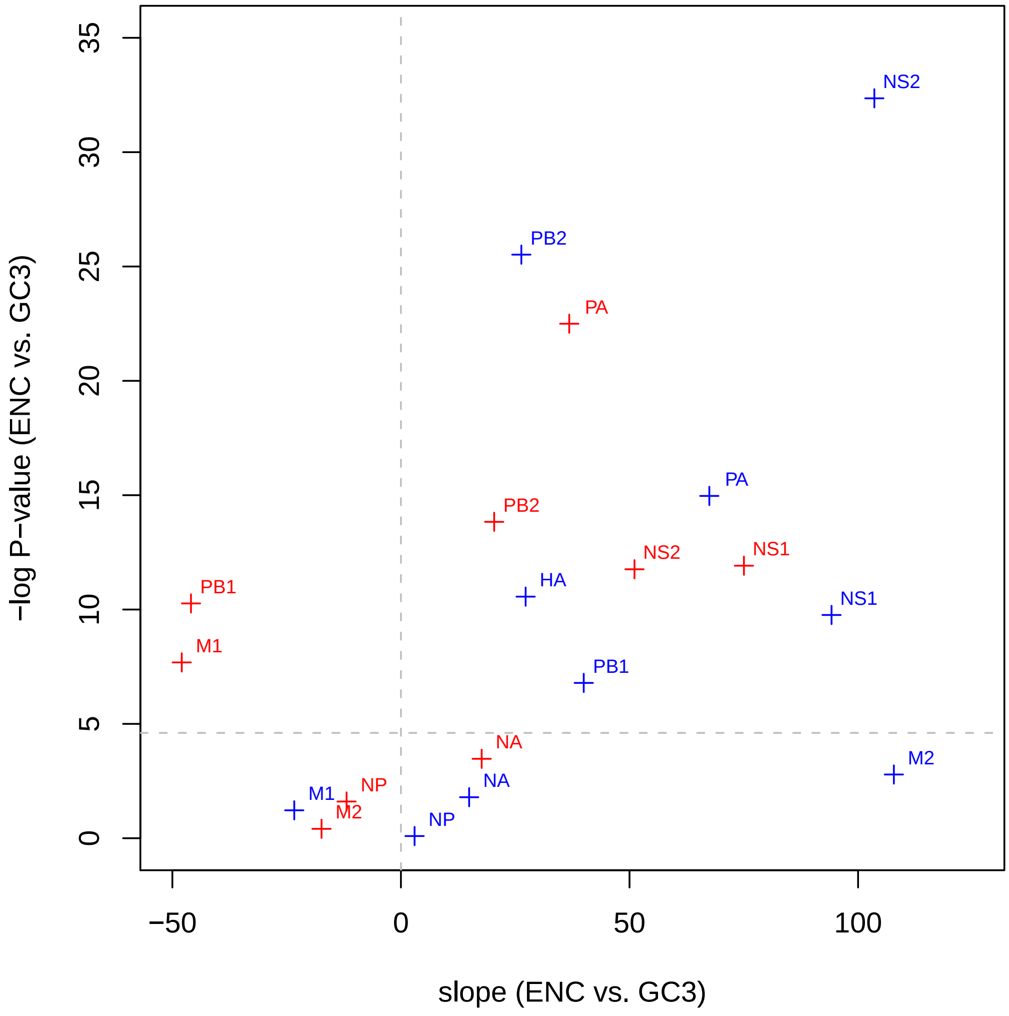


Figure S25: **Significance of the linear fit between effective number of codons and GC3 content.** H1N1 viruses are in red; H3N2 viruses are in blue. The significance level is taken at 1% (dashed horizontal line in gray). HA in H1N1 has a $P$-value of 0, and therefore does not appear on the graph.

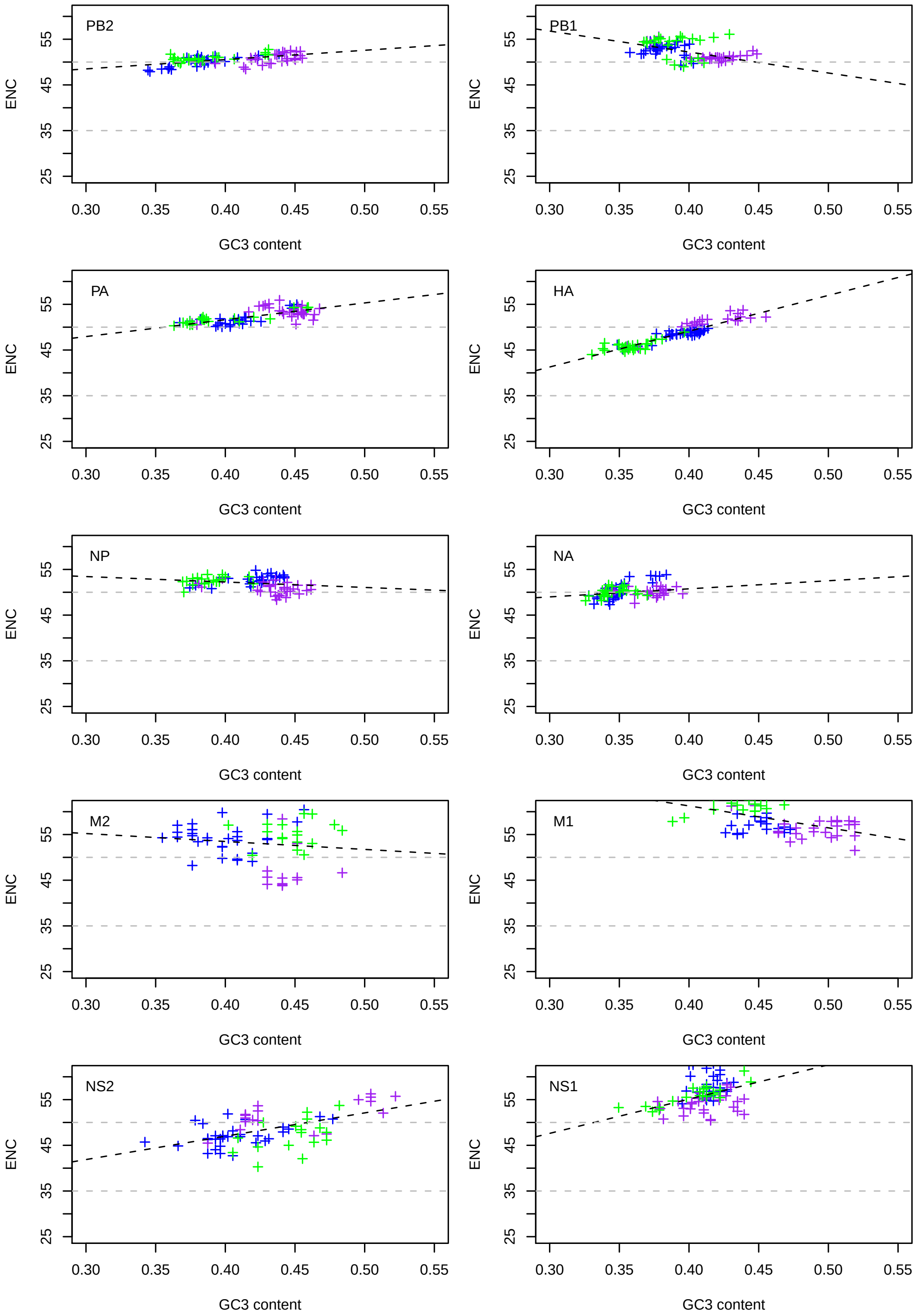


Figure S26: **Effective number of codons (ENC) as a function GC3 content for H1N1 viruses.** Hosts are color-coded: avian in purple, human in blue and swine in green. Gray horizontal lines represent ENC cutoffs at 35 and 50. The linear fit is represented as a black dashed line.

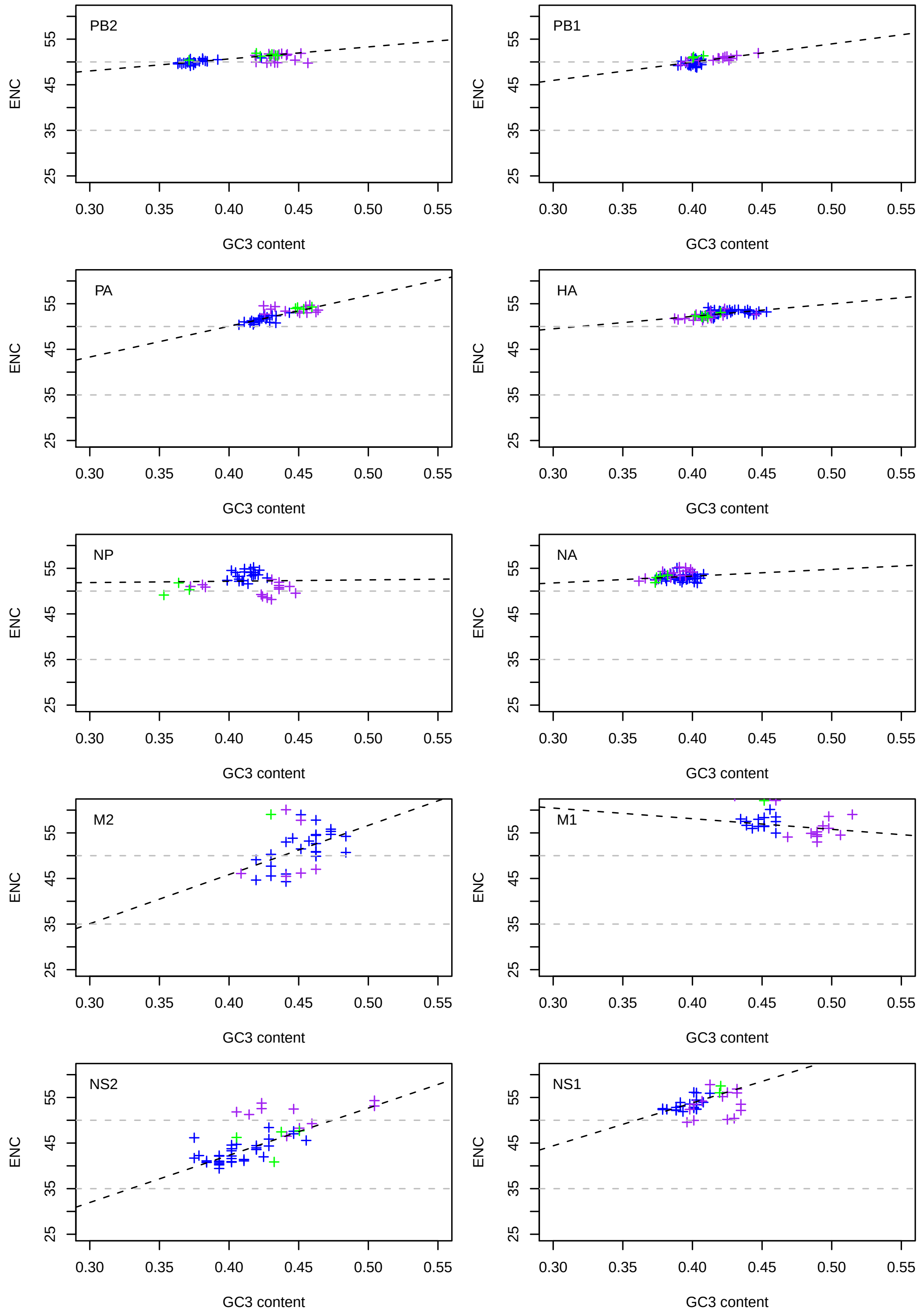


Figure S27: **Effective number of codons (ENC) as a function GC3 content for H3N2 viruses.** Hosts are color-coded: avian in purple, human in blue and swine in green. Gray horizontal lines represent ENC cutoffs at 35 and 50. The linear fit is represented as a black dashed line.

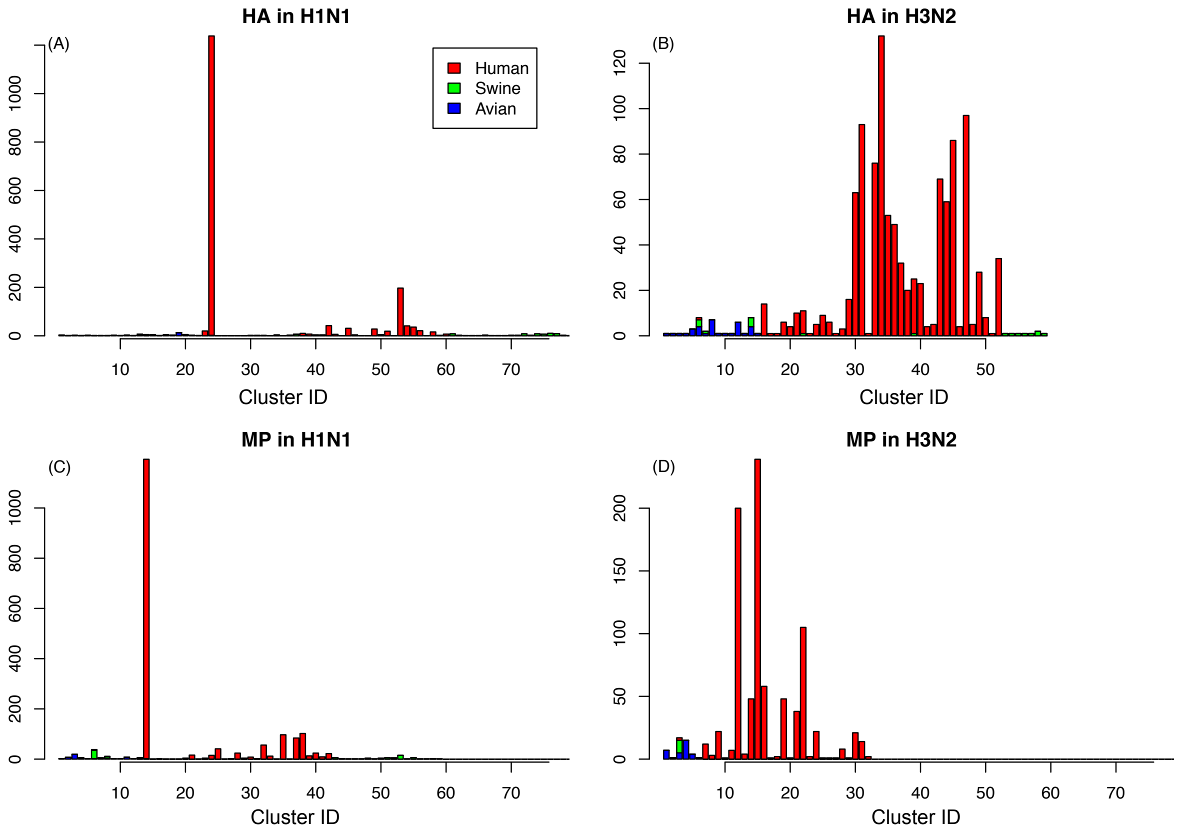


Figure S28: **Cluster composition for select H1N1 and H3N2 genes.** Results of sequences clustering are shown for the fastest evolving gene, HA, for (A) H1N1 and (B) H3N2 viruses as well as for the slowest evolving gene, M1, for (C) H1N1 and (D) H3N2. Hosts are represented in red (human), green (swine) and blue (avian). Cluster IDs are arbitrary; their largest number represent the final number of sequences in the dating analyses.
